# Physics-Based Solubility Prediction for Organic Molecules

**DOI:** 10.1021/acs.chemrev.4c00855

**Published:** 2025-07-29

**Authors:** Daniel J. Fowles, Benedict J. Connaughton, James W. Carter, John B. O. Mitchell, David S. Palmer

**Affiliations:** ‡ Department of Pure and Applied Chemistry, 3527 University of Strathclyde, Glasgow, Scotland G1 1XL, U.K.; ¶ EaStCHEM School of Chemistry and Biomedical Sciences Research Complex, 7486 University of St Andrews, St Andrews, Scotland KY16 9ST, U.K.

## Abstract

Accurate prediction of aqueous solubility for organic molecules is of great importance across a range of fields, from the design and manufacturing of energy materials, to assessing the environmental impact of potential pollutants. It is of particular significance to the pharmaceutical industry, in which problems with low aqueous solubility frequently hamper the development of new drugs. Experimental measurements of solubility are used extensively, but are often time-consuming, resource intensive and only applicable to already synthesized molecules. As such, there is a need for the development of computational approaches to predict solubility. In recent years, there have been considerable advances in physics-based methods, with several contrasting techniques able to give accurate predictions of solubility and a wealth of thermodynamic data for structural optimization. Here, we provide the reader with a thorough understanding of the theoretical background and practical applications of these physics-based methods to predict solubility. This includes discussions of the various advantages and disadvantages of each approach, and an indication of areas of continuing research. Experimental and data-driven methods to assess solubility are also discussed to provide context.

## Graphical abstract

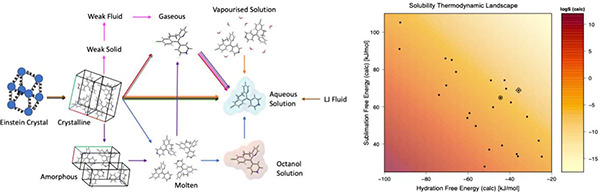

## 1 Introduction

### 1.1 Importance of Solubility

Solubility is a fundamental physicochemical property that is important in many areas of academic and industrial chemical research. Knowing how much of a solute will dissolve in a solvent, and how that value can be modulated by changes in structure, solution composition, or environmental factors, is valuable at all stages of discovery, development, and production of new chemical entities. A case in point is the pharmaceutical industry, where it is estimated that 70% of candidate molecules in development have solubility issues, which have directly contributed to the slowdown in the production of new drugs..^1,2^ Similar issues are observed in other industries. For example, solubility directly impacts the effectiveness, safety, and environmental behavior of agrochemicals like pesticides and herbicides.^3,4^ For chemical manufacturing, where solubility can cause problems with synthesis, purification and characterization of molecules, understanding solubility behavior can be invaluable for process optimization.

Since experimental measurements of solubility are often time-consuming, resource intensive, and only applicable to already synthesized molecules, there is a demand for accurate methods to predict solubility, to be used to guide the selection of solutes, solvents, or environmental conditions, thereby lowering costs and time spent on unnecessary synthesis and testing.^5–8^ Predictive methods can also help to understand the structure–property relationships that determine solubility, thereby aiding molecular design.

The prediction of solubility from molecular structure has been an active area of academic research in theoretical and computational physical chemistry for over a century, from early structure–property relationships, to semiempirical models, to physics-based approaches, to deep learning algorithms. The majority of predictive methods are data-driven approaches, which use statistical models to learn a relationship between the physical property of interest (i.e., solubility) and an appropriate computational representation of the molecule. Although these methods are convenient to use and fast, most are only able to make predictions at fixed environmental and chemical conditions, and are often unreliable for molecules dissimilar to those in their training set. Furthermore, as these methods are not based on any fundamental chemical theory, little information is provided about the underlying chemistry, and as such they are difficult to systematically improve.

The calculation of the solubility of crystalline organic molecules from theory and simulation without parametrization against empirical solubility data has been a longstanding goal in computational chemistry. It has proven to be a significant challenge because of the need to accurately simulate both the solid crystalline and dissolved solution phases, while accounting for a variety of factors that influence the solubility, including crystalline polymorphic form. One significant advantage of physics-based methods is that they offer a more rigorous approach for predicting aqueous solubility than data-driven methods, with a clearly defined theoretical basis, which means they can provide a wealth of structural and thermodynamic data for chemical and process optimization. A considerable number of approaches have been proposed, with significant progress in modeling solvation^9,10^ and simulating crystalline polymorphs^11–13^ for organic molecules having helped contribute to the success of a wide range of them.

This review will provide the reader with a thorough understanding of the theoretical background and practical applications of these physics-based methods to predict solubility. This will include a discussion of the various advantages and disadvantages of each approach, and an indication of areas of continuing research. In Section 1, fundamental concepts are introduced and methods for measuring experimental solubility are reviewed, with particular focus on how the variability in experimental data influences the development and validation of computational methods. To provide context, Section 2 presents the current state-of-the-art in data-driven methods to predict solubility, from traditional QSPR approaches to deep learning. In Section 3, physics-based methods to predict solubility are reviewed. The focus is on methods that do not require parametrization against solubility data, but some semiempirical methods are included where they have a strong theoretical background. Guided by the current literature, Sections 2 and 3 primarily focus on the prediction of intrinsic aqueous solubility, but in Section 4 the scope is broadened to consider prediction of solubility in other solvents and under other conditions. Sections 5 and 6 provide Conclusions and Future Perspectives that summarize recent progress and highlight promising avenues for further work. The scope of the review encompasses crystalline small organic solutes, including druglike molecules and other small functional organic molecules, but not large polymers, which often require different computational methods to model accurately. The review begins in the next section with a description of the relevant theoretical background and key concepts.

### 1.2 Solvation, Dissolution, and Solubility

Solvation describes the process through which a dissolved substance and solvent interact. As a solid solute dissolves, particles will shift from the bulk solid to solution with the formation of solute–solvent complexes, and an equilibrium will emerge with dissolution and precipitation as opposing processes. If the amount of solute added to the solution exceeds the solubility limit, dissolution will continue until the concentration of dissolved solute exceeds that which can be favorably held in solution (“supersaturation”), at which point the rate of precipitation will exceed the rate of dissolution and dissolved solute will revert to the bulk solid. Solubility represents the point at which a stable solute–solvent thermodynamic equilibrium has been achieved, with the rate of dissolution equal to the rate of precipitation. The solute and solvent of a binary mixture have the same chemical potential at thermodynamic equilibrium and are in coexistence with each other. From this, solubility can be generally defined as the maximum amount of substance that can dissolve in a specified amount of solvent at thermodynamic equilibrium under specified environmental conditions.

### 1.3 Factors That Influence Solubility

The solubility of a compound can be influenced by many factors, ranging from changes in environmental conditions to the introduction of new chemical species. A selection of these factors will be discussed over the next few sections in order to provide relevant background for the discussion of computational methods to predict solubility.

#### 1.3.1 Temperature

The solubility of a solute in a solvent is a function of the temperature. The solubility of a compound will shift depending on whether the enthalpy change of the dissolution reaction is endothermic (Δ*H*_*dissolution*_ > 0) or exothermic (Δ*H*_*dissolution*_ < 0). For most solid compounds, a higher temperature will further induce a breakdown of its crystal lattice, resulting in greater mobility from the bulk to solution, and increased solubility. There are exceptions, such as sodium sulfate, which forms less soluble hydrate complexes at higher temperatures.^14^ Gaseous compounds typically trend in the opposite direction to solid species, with higher temperatures leading to greater degasification of the solvent. Changes to temperature and dissolution enthalpy can be related to a change in solubility through the van’t Hoff equation.^15^

1 $$\ln\frac{K_{2}}{K_{1}}=\frac{\Delta_{r}H^{⊖}}{R}\left(\frac{1}{T_{1}}-\frac{1}{T_{2}}\right)$$

where *K*_1_ and *K*_2_ are the solubility products at temperatures *T*_1_ and *T*_2_, respectively, Δ_*r*_H^⊖^ is the standard dissolution enthalpy and *R* is the molar gas constant. The solubility product defines the concentration of dissolved species in a saturated solution. The van’t Hoff equation lacks solvent specific expressions, and so will struggle to predict solubilities across different solvents.

The linear form of the van’t Hoff equation can be used to determine whether a compound will exhibit a higher or lower solubility by estimating the enthalpy and entropy of dissolution.^16,17^ By measuring the solubility product at different temperatures, the change in enthalpy can be used to ascertain the endothermic or exothermic nature of the species.

2 $$\ln K_{sp}=-\frac{\Delta_{r}H^{⊖}}{RT}+\frac{\Delta_{r}S^{⊖}}{R}$$

where *K*_*sp*_ is the solubility product at a given temperature and Δ_*r*_S^⊖^ is the standard dissolution entropy.

The van’t Hoff equation includes an implicit assumption that enthalpy and entropy do not change with temperature. This assumption does not hold for all species, and so an additional term must be introduced to correct for this.

3 $$\ln K_{sp}=a+\frac{b}{T}+\frac{c}{T^{2}}$$

where

4 $$\Delta_{r}H=-R+\left(b+\frac{2c}{T}\right)$$

and

5 $$\Delta_{r}S=R+\left(a+\frac{c}{T^{2}}\right)$$

#### 1.3.2 Pressure

The effects of pressure on solubility only needs to be considered for gaseous species. The pressure dependence for condensed phases is often insignificant and can be neglected in practice. Gaseous solutes form an equilibrium between gas present above the solvent and that which has dissolved within the solvent. The solubility product, and so the solubility of a given gaseous compound, can be quantified with Henry’s law.^18^

6 $$\rho =k_{H}c$$

where ρ is the partial pressure, *k*_*H*_ is the Henry’s law constant for a given gaseous solute and *c* is the concentration of dissolved gas in solution. Henry’s law states that the concentration of dissolved gas in solution is directly proportional to the gaseous partial pressure above the solution. By altering the pressure of a system, the partial pressure will be directly altered. An increase in pressure will cause the gas above the solvent to compress, leading to an increase in partial pressure and so greater solubility. Henry’s law is only an approximation for systems where the gaseous species is present at a low concentration, and in solutions where no chemical reaction is taking place.

#### 1.3.3 pH and Buffers

A commonly applied and effective technique for adjusting the solubility of an ionizable solute within a given solvent is by altering the pH of that medium. The relationship between bulk solution pH and the extent of solute solubilization depends upon the number and identity of charged groups found within the solute structure. For example, the solubility of compounds containing basic anions can generally be increased through a reduction in solvent pH. From the addition of an acidic compound, newly introduced H^+^ ions will react with basic anions present in solution to form water. H_3_O^+^ ions will also form from the reaction of H^+^ ions and water. This shift in equilibrium will thus increase the solubility of the bulk compound.

7 $$\mathrm{Mg}{\left(\mathrm{OH}\right)}_{2\left(s\right)}⇌{\mathrm{Mg}}_{\left(\mathrm{aq}\right)}^{2+}+2{\mathrm{OH}}_{\left(\mathrm{aq}\right)}^{-}$$

8 $$H_{\left(\mathrm{aq}\right)}^{+}+{\mathrm{OH}}_{\left(\mathrm{aq}\right)}^{-}\to H_{2}O_{\left(l\right)}$$

Buffers are one method used extensively to control the solution pH. A buffer solution is any aqueous based mixture of compounds containing a weak acid and its conjugate base, or vice versa. The purpose of a buffer solution is to minimize any change to the pH of the solution when a strong acid or base is added. Buffer solutions are able to maintain a stable pH because of the chemical equilibrium that forms between the weak acid and its conjugate base. This equilibrium will help to control the concentration of H^+^ and H_3_O^+^ ions present in solution as a strong acid or base is added. No single buffer is effective over the full pH range, and multiple buffers may be needed to obtain pH control over a wide range. For example, a combination of phosphate and citrate is very effective as a buffer over a pH range of 1.6–7.7, and acetic acid acts as a buffer in a pH range of 3.7–5.8 (shown below).

9 $${\mathrm{CH}}_{3}{\mathrm{COOH}}_{\left(\mathrm{aq}\right)}+H_{2}O_{\left(l\right)}⇌{\mathrm{CH}}_{3}{\mathrm{COO}}_{\left(\mathrm{aq}\right)}^{-}+H_{3}O_{\left(\mathrm{aq}\right)}^{+}$$

In solution, acetic acid will dissociate into acetate and H^+^ ions. If a strong acid is added, then the presence of existing H^+^ ions will limit the increase in hydrogen ion concentration, and instead the equilibrium position will shift toward the weak acid. Similarly, adding a strong base will not raise the pH as OH^–^ ions will instead react with the weak acid.

The Henderson–Hasselbalch equation can be used to estimate the pH of a buffer solution from the concentrations of weak acid and conjugate base in solution.^19,20^

10 $$\mathrm{pH}={\mathrm{pK}}_{a}+\log_{10}\left(\frac{\left[\mathrm{Base}\right]}{\left[\mathrm{Acid}\right]}\right)$$

where p*K*_a_ is the acid dissociation constant and the terms in square brackets denote the concentration of conjugate base and weak acid, respectively.

#### 1.3.4 Cosolvents, Surfactants and Complexation

Many classes of nonpolar compound exhibit poor aqueous solubility and poor miscibility with water. This observed tendency of nonpolar substances to aggregate in an aqueous solution and to be excluded by water is sometimes referred to as the hydrophobic effect. The origin of the hydrophobic effect is still debated,^21–25^ but it arises at least in part from the inability of nonpolar compounds to form strong hydrogen bonds with water. In pure water, there is a dynamic network of hydrogen bonds between solvent molecules. The enthalpic change for hydrating a solute is dominated by an unfavorable contribution for creating a cavity in the solvent for the solute, and favorable contributions from solute–solvent interactions, the latter of which are typically bigger for hydrophilic than hydrophobic compounds. Close to the surface of the solute, hydrogen bonds reorientate forming a slightly more ordered ”cage” around some solutes, with a corresponding loss of translation and rotational entropy of the solvent molecules. The hydrophobic effect is observed when the interplay between enthalpic and entropic factors makes solvation less favorable than aggregation. Aggregation of nonpolar compounds occurs because it reduces the water-accessible surface area, thereby minimizing the disruptive effect.

If the compound under investigation is a weak electrolyte, then altering the pH of the solution may be sufficient to solubilize it, but for many classes of compound a change in pH is not sufficient, and so other techniques must be applied to counteract this behavior.^26^ Many such techniques make use of hydrotropes to solubilize hydrophobic compounds, of which three such approaches will be briefly discussed below. Interested readers are directed toward more in depth reviews covering these topics.^27,28^

Cosolvents are organic compounds that can be introduced to an aqueous solution to better match the polarity of water with that of the poorly soluble solute. These compounds typically maintain a mutual miscibility with water through the presence of hydrogen bond donor and/or hydrogen bond acceptor groups. Cosolvents typically contain a small hydrocarbon region, reducing the hydrogen bond density within its vicinity. This nonpolar pocket provides space for a previously poorly soluble solute to exist in solution and thus improve its solubility. As the introduction of a nonpolar cosolvent will reduce the polarity of water, improvements in solubility will only be observed for nonpolar solutes, with a reduction in solubility for most polar compounds. A wide range of cosolvents are in common use, and studies directly investigating new cosolvent mixtures, as well as their application in *in-silico* methods, are regularly released.^26,29–32^

Surfactants are amphiphilic molecules, characterized by the presence of both hydrophilic and hydrophobic groups. These opposing groups drive a tendency for amphiphilic molecules to orient themselves at the interface between phases of different polarity. For multiphase systems involving water, the surfactant will orient itself with its more polar region directed toward water and its more nonpolar region toward the less polar phase. As an aqueous solution becomes saturated with surfactant, groups of surfactant molecules will aggregate to form micelles. These typically spherical structures maximize the favorable interactions between the hydrophilic head of each individual surfactant and water while maintaining a nonpolar core. Beyond macroscopic interfaces, surfactants will also orient themselves to the microscopic interface between a solute and water. The micellar surfactant will incorporate the solute within its structure, with the exact location dependent on the polarity of the solute. The greater the difference in polarity between the solute and water, the more likely it is that the solute will be incorporated closer to the core of the micelle. Surfactant micelles provide nonpolar solutes with a favorable position within solution, thus improving their solubility. Improvements in the solubilization of poorly soluble drugs in water are often reported, such as those reported for erythromycin with the inclusion of nonionic surfactants by Bhat et al.,^33^ as well as for systems in which solubility is limited by pH.^34,35^

Solubilisation by complexation is a common method for improving the solubility of poorly soluble compounds. Complexing agents work similarly to surfactants, by creating a favorable intermolecular association between water-soluble ligands and poorly soluble substrates within an aqueous environment.^36^ There are multiple classes of complexing agent actively used to improve the solubility of organic molecules, including metal coordination complexes, organic molecular complexes, inclusion complexes and pharmacosomes.^36^ Coordination complexes, unlike other classes of complexing agent, form through the covalent bonding of ligands with a central metal cation. The formation of water-soluble metal complexes has been shown to improve the solubility and stability of a variety of commercial drugs.^37,38^ The complexation of organic substrates within organic, inclusion and pharmacosome complexes occurs through a range of noncovalent interactions, such as hydrogen bonding and charge-transfer. Organic molecular complexes are often formed between a poorly soluble drug molecule and a similarly sized ligand or polymer. The introduction of caffeine^39^ or β-cyclodextrin^40^ to an aqueous solution of sulfathiozole leads to a significant increase in the solubility of sulfathiozole. Polyvinylpyrrolidone is a very common polymeric complexing agent, and has been shown to improve the solubility of a range of drug molecules.^41,42^ Inclusion complexes can be generally defined as host–guest complexes, where the substrate is partially or fully enveloped within the ligand superstructure. Several variations of these ligands exist, such as clathrates, channel lattice complexes^43^ and supramolecular cyclic structures.^44^ Pharmacosomes, which possess the most similarity to surfactants, are amphiphilic phospholipid structures that form concentric lipid bilayers within aqueous solution. Substrate-phospholipid complexes can increase substrate solubility in both aqueous and nonaqueous solutions.^45,46^

#### 1.3.5 Solid Form and Solubility

Observed solubility values will be dependent on the physical form of the precipitate. If the precipitate is a solid crystal, then the crystalline polymorphs must be considered. Many, perhaps even most, organic compounds are capable of crystallizing into two or more polymorphs with distinct crystal structures. Since by definition it has the lowest free energy, the most stable polymorph is always anticipated to be the least soluble solid form.

Kinetic solubilities can be assigned to any polymorph that is not the most thermodynamically stable crystalline form of the solute. Such polymorphs form according to Ostwald’s rule,^47^ which states that less stable polymorphs precipitate more rapidly due to their kinetically favorable disorganized state. Given enough time, kinetic polymorphs will rearrange into more thermodynamically stable states, until the crystal structure with the lowest free energy is found. A kinetic solubility value can typically be assigned to any metastable polymorph, provided it is stable enough to exist as the precipitate without changing form. The precipitate observed is strongly dependent on the conditions and method used to measure solubility and is unlikely to be reproduced under different protocols.^48^ Any combination of amorphous, crystalline, salt or cocrystal states could contribute to the physical make up of the precipitate. Typically, solubilities of polymorphs of the same compound differ by factors of two or less, which corresponds to about 1.8 kJ mol^–1^ at room temperature, though occasionally these differences may be of an order of magnitude or more.^49^

Beyond polymorphic crystal forms of the compound itself, there may also be hydrates, solvates or cocrystals with other species present in the crystal.^50^ The existence of different possible solid forms raises questions both of the identity of the solid present in a given solubility experiment, and also of whether the most thermodynamically stable polymorph of the given compound has even been discovered yet.^51^ The nature of the solid present may depend on the solubility technique being employed, for instance dispensing from DMSO typically leads to amorphous solids, while potentiometric methods allow more control. In a particularly impressive example of the latter, Llinas et al.^52^ were able to discover a new stablest polymorph of the pharmaceutical compound sulindac, and to measure distinct solubilities for both polymorphs in the same experiment.

### 1.4 Definitions of Solubility

The many factors that influence solubility have given rise to different definitions of solubility in the published literature. For the purposes of discussing the physics-based (and data-driven) predictive methods in this field, the most useful definition to start from is ”intrinsic solubility” because it is clearly defined, can be measured accurately, and has been widely adopted in modeling studies, including two community-wide blind challenges.

#### 1.4.1 Intrinsic Solubility

Intrinsic solubility is defined as the concentration of the un-ionized form of an ionizable molecule in a saturated solution at thermodynamic equilibrium at a given temperature. Several different models make use of intrinsic solubility for the determination of molecule specific solvated behavior. One such model is the Noyes-Whitney equation, used to calculate dissolution rate.^53^

11 $$\frac{dW}{dt}=\frac{DA\left(C_{s}-C\right)}{L}$$

where the left-hand term describes the rate of dissolution, *D* is the diffusion coefficient specific to a given solute–solvent pair, *A* is the surface area of the crystalline solute, *C* is the concentration of the bulk solid, *C*_*s*_ is the concentration of the solute in solution and *L* is the diffusion layer thickness.

The Noyes-Whitney equation accurately describes the relationship between dissolution rate and solubility, with a poor dissolution rate often mirrored by an equally poor solubility, and vice versa. Many parameters influence a solute’s dissolution rate, and cause the rate to change as dissolution occurs. For example, the particle surface area of a solute does not typically remain constant during dissolution, often decreasing in size over time. This results in a dissolution rate which similarly decreases with time, thus influencing any measurements taken later.

### 1.5 Experimental Methods for Intrinsic Solubility Determination

The shake flask method^48,54^ is a conceptually simple approach to solubility determination, the principle being simply to keep dissolving solute in the solution until it is saturated. Thus, an excess of sample is added to the solution, which often includes a buffer, and the sample is shaken at a given temperature and pressure to dissolve as much sample as possible. This is expected to yield a supersaturated solution, and the solution will require a period of time to reach equilibrium, the length of which may be difficult to estimate. Excess sample is filtered off, and the concentration of the remaining equilibrated solution is measured; usually by a process involving liquid chromatography linked to detection by either UV/visible spectroscopy or mass spectrometry.

Solvent evaporation methods such as LYSA^48,55,56^ have some similarities with the typical industrial implementations of the shake flask approach, the major difference being that the sample is typically delivered as a solution in an organic solvent, which is then evaporated to leave a solid sample, not necessarily crystalline. Again, spectroscopic or chromatographic methods can be used to measure the saturated concentration, calibration to known concentrations of the stock solution facilitating quantitative results.

CheqSol (Chasing equilibrium Solubility)^52,57–59^ is a potentiometric titration approach applicable to compounds with a titrable ionizable group, whether acidic or basic. The procedure uses automated acid–base titrations to shuttle between supersaturated and subsaturated concentrations a little higher and lower than that at which precipitation is observed. In this way, the system is forced toward equilibrium on a time scale of tens of minutes to around an hour. The continued cycling between super- and subsaturated solutions has been used to identify changes in the crystalline polymorphic form of the precipitate in some cases.^52^ While CheqSol remains a low-throughput method, it avoids long equilibration times and uncertainty over whether equilibrium has been reached. Similar to CheqSol, the Dissolution Template Titration (DTT) method is also a potentiometric approach.^60^ In DTT, the problem is understood in terms of a three-state model, consisting of an initial state before titrant addition, an intermediate state following titrant addition but prior to solute dissolution, and the final equilibrium state. The equilibrium solubility is calculated by analyzing the Bjerrum plot for the titration under the assumption that this three-state model applies.^60^

The synthetic method^54,61,62^ uses the intensity of a laser passed through a solution to assess the presence of solid matter. The intensity is measured first for pure solvent, then a small quantity of solute is added. The detected laser light intensity will initially drop, but should recover as the solid is dissolved. Saturation is indicated by the lowest concentration at which the signal fails to recover full intensity after addition of a further small quantity of solid. The same approach can be used to measure temperature-dependence of solubility, by varying the experimental temperature and finding the limit where full dissolution no longer occurs at a known concentration.

A contrasting approach is to adapt the high-throughput kinetic solubility assays commonly used by pharmaceutical companies, in the hope of tweaking them to facilitate equilibrium solubility determination. These methods, however, generally rely on compounds being delivered from stock solutions in dimethyl sulfoxide (DMSO), which means that the assayed solution will contain some DMSO effectively acting as a cosolvent. The method is likely to overestimate the equilibrium solubility due to remaining supersaturation and the experimental time scale would also need to be extended to at least several hours.^48^

While all these methods are widely used, each of them has distinct advantages and disadvantages. The shake flask method is the traditional gold standard, offering reliable and accurate thermodynamic solubility measurements. However, it is time-consuming, labor-intensive, and requires a significant amount of material, making it less suitable for early stage drug discovery. Solvent evaporation methods, such as LYSA, provide rapid results using minimal sample quantities, but evaporation-based techniques can lead to nonequilibrium conditions, potentially skewing results. Potentiometric methods, such as CheqSol and Dissolution Template Titration (DTT), offer fast, automated solubility measurements by tracking pH changes. These methods are convenient for ionizable compounds and can provide insight into both kinetic and thermodynamic solubility, and often additional information about acid–base behavior, but they may not be applicable for nonionizable molecules and require precise calibration. High-throughput methods that involve diluting compounds into aqueous solutions from DMSO stock allow for rapid screening with small amounts of material. However, DMSO can affect the solubility of certain compounds, leading to potential inaccuracies, and the method may not capture the desired solubility accurately due to the presence of the DMSO. The techniques most suited to accurate measurement of equilibrium solubilities, the shake flask and CheqSol methods, require effort of at least the order of hours per compound. Higher throughput assays, on the other hand, are more suited to give only an order of magnitude estimate of the equilibrium solubility, which while useful in some contexts does not yield precise or accurate values. Benchmarking computational chemistry methods is not really possible without the best available experimental data, including an understanding of the magnitude of likely experimental errors.

### 1.6 Errors in Experimental Solubility Data

As noted elsewhere,^63^ various kinds of errors can occur in solubility data, especially secondary data in the literature. The first kind are what have been called gross errors, essentially outright mistakes. Sometimes that might be due to a failure to appreciate the difference between the solubility of the neutral form, as required for intrinsic solubility, and that of ionised acids or bases. Another possibility is that the distinction between kinetic and equilibrium solubilities is blurred, either due to misunderstanding or to a rushed experimental time scale leading to a supersaturated value being reported. The solubility determination of indomethacin, as discussed in detail by Comer et al.,^59^ exemplifies a different type of mistake, where previously researchers had failed to anticipate a chemical reaction occurring under the experimental pH conditions, leading to the solubility of the wrong compound being recorded. A further possibility is typographical errors, which can occur either through mistyping by hand numbers from a literature source, such as writing log S = −3.79 as log S = −7.39, or by accidentally mishandling electronic data.

A second class of error is the systematic error that might occur between sets of solubility determinations which may result from differences in technique or between different laboratories, even if in principle the same technique has been applied. The possibility of repeatable differences between say CheqSol and Shake Flask has been investigated to some extent by Avdeef, see Figure 3 of ref 56. That analysis does not suggest that regressions between methods have gradients differing from unity by much more than 0.01 or intercepts much larger than 0.15 log S units. Distinct laboratories may also have different practices in terms of the extent to which variables such as temperature and pH are controlled and monitored. Similar considerations affect the compilation of solubility data sets and the extent to which variations in reported experimental temperatures or pH are acceptable in data that may later be used as a training or test set for solubility prediction. High-throughput solubility assays based on multiwell plates may have systematic differences first between different plates, and second between the corresponding positions, that is the same row and column numbers, on different plates. The interplate differences are likely to be a simple consequence of each plate often being loaded with a set of similar compounds from a single source, while the location-based differences may be the result of systematic biases in the dispensing or reading technologies, as suggested by Rohde in the context of the *first EUOS/SLAS Joint Challenge: Compound Solubility*.^64^

Third, there are random errors between repeat runs of the same experiment as conducted by the same experimenters with the same apparatus. For CheqSol, this kind of reproducibility error is asserted to be as small as ± 0.05 log S units.^65^ Small random differences between runs are unavoidable, but their effects can be reduced and mitigated by running multiple repetitions; albeit with a consequential reduction in throughput.

Additional factors may cause errors in solubility assays. A significant fraction of small organic molecules with low aqueous solubility, including some marketed drugs, can form nano- or microscale agglomerates (30 nm to 1000 nm diameter).^66^ These colloidal aggregates can skew experimental solubility results, leading to under- or overestimated solubilities.^67^ Separately, they also cause problems for some biological assays, where unspecific binding of the aggregate to proteins can cause local denaturation and apparent inhibition.^68^ Algorithmic methods have been developed to identify self-aggregation behavior based on either QSPRs or chemical similarity to known aggregators, though the latter approach is limited by design to known chemotypes. Key descriptors in these models normally capture aspects of hydrophobicity (e.g., logP or hydrophobic surface area), hydrogen bond donor/acceptor characteristics, and molecular structure (e.g., no. of sp^3^ centers, self-complimentarity between key interaction sites), but individually few of these descriptors are specific to the aggregation process.^68,69^ The models that have been developed to predict self-aggregation can be used as computational filters to remove molecules that are likely to cause problems in experimental assays. When combined with computational solubility predictions, this makes it possible to prioritize for experimental analysis those molecules that have the desired solubility profile and are expected to be easier to analyze. The inclusion of ”self-aggregation” descriptors in data-driven solubility models would not be expected to lead to an improvement in performance because the descriptors are not specific to the aggregation process, as noted above.

One of the most significant works discussing the size and nature of errors in solubility data is by Avdeef.^56^ He demonstrates that, by very careful and time-consuming curation of the data, common sources of error can be identified, accounted for, and in some cases corrected. This requires critical analysis and detailed understanding of the experimental procedures used, thus identifying instances where the experimental conditions such as pH, buffers, temperature, technique, purity, crystallinity of the solid and compound stability may require accounting for. By this kind of careful and thorough approach, Avdeef and colleagues were able to prepare the 100-compound ‘tight’ data set for the 2019 Solubility Challenge,^5^ with a claimed interlaboratory standard deviation as low as ± 0.17 log S units, a far cry from more commonly cited figures in the region of 0.5–0.7 log *S* units.^56^ This required data selection as well as data curation and correction. An accompanying 32-compound ‘loose’ data set contained compounds where the data could not be corralled into such close alignment, and had a cited interlaboratory standard deviation of ± 0.62 log S units.

Since there is some uncertainty as to how large the errors in experimental solubility data are, it is therefore not clear exactly how much of the observed RMSE of a predictive method is down to experimental error and how much is due to the limitations of predictive models. In practice, teasing these components apart may be problematic, since experimental error impacts the prediction process twice for data-driven models; once in the training data on which the model is built, and a second time in the test data against which the predictive performance is assessed.

## 2 Data-Driven, Machine Learning, and AI-Based Methods

Recent advances in data-driven modeling and machine learning have had significant impacts on many fields, including chemistry. However, attempts to find quantitative relationships between chemical structure and experimental properties such as solubility have a long history, an early example being the work of Fühner from 1924 in which the solubility of a series of hydrocarbons was related to the number of methylene groups.^70^ Development of Quantitative Structure Property Relationship (QSPR) modeling in the 1960s by Hansch,^71^ provided a new approach for building predictive models and although initially used to predict biological activity, these techniques were quickly applied to the prediction of other ADMET and physicochemical properties. Many early models were constructed using multilinear regression, similar to the group contribution approach, but approaches have since been expanded to other methods, including Support Vector Machines (SVM), Random Forests (RF) and other tree based methods and Neural Networks (NN),^72,73^ benefiting from the advances in machine learning during the 1990s and 2000s.

More recently the advent of deep learning has brought with it expectations for another step-change in prediction accuracy. However, while deep learning has shown dramatic advances in many fields, including in chemistry with the AlphaFold2 model for protein structure prediction,^74^ a similarly significant improvement has not yet been demonstrated in property prediction. These techniques bring with them a number of new challenges, particularly the need for large data sets with reliable and consistently measured solubility values. Outstanding questions also remain on the best way to represent molecules within deep learning, with competing descriptor, graph and text based approaches, and use of 2D or 3D information. The more “black-box” nature of deep learning also makes understanding the reasons behind particular predictions significantly more challenging.

A useful measure of the recent progress in data-driven solubility prediction can be made by considering the solubility challenges, the first posed in 2008 ^65^ and the second released 10 years later.^5^ The first Solubility Challenge was proposed to address the large numbers of prediction models published and to try to evaluate the different approaches, however, the results proved inconclusive. In the intervening years, debate has focused on whether the limiting factor is the accuracy and consistency of experimental solubility data or limitations in the methods and algorithms used.^75^ The intervening decade has also seen the wider adoption of machine learning in property prediction and the introduction of deep learning techniques to chemistry and solubility prediction specifically and by the second challenge, in 2018, all models submitted used QSPR or Machine Learning (ML) based models.

### 2.1 Group Contribution and QSPR Approaches

A conceptually simple way to predict various properties from molecular structure is the Group Contribution (GC) approach, in which the property of interest is predicted based on the presence and counts of certain molecular substructures or functional groups. In its simplest form, the GC approach uses a weighted sum over groups and its success relies on the additivity of the property being predicted. An early application to solubility by Klopman et al.,^76^ built on earlier work to predict log *P* resulting in an RMSD of 1.25. This work was further developed using an expanded fragment set reducing the RMSD to 0.84 on a larger 120 compound test set.^77,78^ Hou et al., found that an atom, rather than fragment, based approach gave further improvement with an RMSD of 0.79 on the same 120 compound test set^78^ with the added advantage that atom based approaches are not necesserily limited to a specific region of fragment space.

Group contribution based methods can work well within a series of related molecules or a limited region of chemical space, but suffer limitations when applied to molecules dissimilar to the training set, especially if new fragments are present. Multilevel GC methods go some way to accommodating this and also capturing collective or proximity effects. By including second and third degree corrections Marrero et al., were able to achieve an accuracy of 0.55 log units.^79^

While the GC approach is relatively successful in capturing additive behavior in the liquid phase, both for solubility and other related properties, such as log *P*,^77^ it is less able to capture the complexity, nonadditive behavior and long-range interactions associated with the crystal phase, a considerable limitation for solid solubility prediction. GC solubility models generally perform better on liquids than solids and additional descriptors or experimental data such as melting points are often needed to improve the treatment of the solid phase.^80^

GC methods are also used to model components in other solubility prediction approaches. For example, GC based log *P* prediction models^81^ are important chemical descriptors for QSPR models and have been used as a substitute for experimental data in semiempirical methods such as the General Solubility Equation.^82^

QSPR modeling can be considered as an expansion on the group contribution approach. The simplest models are based on multilinear regression, mirroring group contribution models, but with variables which indicate the presence of specific functional groups replaced with a broader set of descriptors or molecular fingerprints. Thousands of different descriptors exist, with many freely available in open-source chemoinformatics toolkits.^83^ Descriptors include familiar molecular properties such as molecular weight, charge and numbers of hydrogen donors and acceptors, but also values calculated from the topology and connectivity of the molecule to capture the 2D structure. In some cases descriptors are also calculated from 3D conformations.

The ESOL method developed by Delaney^84^ uses 9 descriptors derived from chemical formula or 2D chemical structure and fit to 2874 experimental intrinsic solubility values. The model achieves a test set RMSD of 1.01, which represents a similar performance to the GSE, but unlike the GSE, ESOL contains no experimental parameters. Huuskonen on the other hand, used a set of more abstract descriptors including E-state indices to fit a NN with a single hidden layer.^85^ The model performed well on a test set spanning the same chemical space as the training set with RSMD of 0.6. Both of these models and the associated data sets have become important benchmarks and are often used to assess the performance of new approaches.

Descriptors can also be developed from separate computationally calculated chemical properties and used as input to a QSPR model to introduce additional physics-based information, such as through the use of molecular simulation. Jorgenson and Duffy^86^ used Monte Carlo simulations to calculate interaction energies and molecular surface areas, averaged over 3D conformations. The resulting model contained only 5 descriptors in total, but gave an RMSD of 0.55, comparable to or better than many GC based approaches. Although supplementing QSPR models with data from alternative computational approaches is attractive, the disadvantage of these more complex descriptors is the additional steps and time needed when making predictions on new compounds.

### 2.2 Deep Learning

In the last 10 to 15 years, the accumulation and availability of huge data sets alongside increases in hardware performance, specifically the use of GPUs, has initiated the field of deep learning. The importance of this field was recognized with the award of the 2018 Turing Prize and in many areas has led to a significant jump forward in predictive power and accuracy, with landmark achievements made in areas including image recognition (AlexNet),^87^ and protein structure prediction.^74^ Deep learning shows huge promise and potential and is being applied to a range of prediction tasks within chemistry. However, outstanding questions such as how to represent molecules and optimal model architecture remain.

#### 2.2.1 Deep NNs

Deep learning methods are based on neural network models. However, the first uses of neural networks for solubility prediction predate the recent explosion in interest. In 1991, Bodor et al.^88^ compared the performance of a NN and simple regression models, finding that the neural network based model outperformed the simple model even on a small data set of 300 compounds. A more recent study from Boobier et al., also found that a NN with a single hidden layer outperformed other models on a 100 molecule data set.^89^ However, whereas Bodor’s model contains 18 neurons, typical deep learning models contain thousands or millions of fitted parameters and often multiple hidden layers, thus requiring much larger training data sets.

A number of deep NNs trained on molecular descriptors or fingerprints, similar to QSPR models, have been developed. Conn et al. looked at the effect that training data set size and different descriptor sets have on model performance and highlighted the importance of not just the size of the training data set, but also the chemical space represented.^90^ Cui et al. investigated the impact of network architecture on performance and specifically the network depth.^91^ Using a ResNet with convolutional layers trained on PubChem molecular fingerprints, they found that very deep networks with 20 or 26 hidden layers were the best performing on their test data.

#### 2.2.2 Graph-Based NNs

Deep learning has also led to new architectures and new approaches allowing learning directly from molecular structures. Graph based NNs have shown promise in chemical applications and intuitively appear well suited to handling 2D molecular structures, removing the need to first represent the molecule numerically using precomputed descriptors. Graph based NNs for chemistry differ from the Graph Convolutional NN (GCNN) in some other fields, such as spectral GCNNs, as they must be able to accept graphs of various sizes and structures. All spatial graph based NNs use the same basic operations and are related to general message passing NNs.^92^ At each layer, information at each node is shared with the neighboring nodes, allowing the influence of each atom in the molecule to gradually spread across the molecule.

Graph based networks were used in one of the early applications of deep learning to aqueous solubility prediction by Lusci et al.^93^ Molecules were represented as a set of directed graphs, in which for each atom in a molecule, there is a separate graph with each bond in the graph pointing toward that one atom. Atoms are represented as feature vectors and information from each atom is mixed with neighboring nodes at each level of the network following the direction of the bonds. The inclusion of additional, physics-based information in the form of a log *P* descriptor was found to have no impact on overall performance for most models. This method was tested on a number of common benchmark data sets, with an average accuracy of 0.58 reported on the ESOL data set and an RMSD of 0.90 on the first Solubility Challenge.

A different approach was taken by Duvenaud et al. to define a different GCNN algorithm.^94^ Molecules were represented as undirected graphs and the graph convolutional layers were used to generate a learnt molecular fingerprint. The model showed comparable performance to Lusci’s model, with an average error of 0.52 when tested on the ESOL data set,^84^ and outperformed other NN models built using precomputed fingerprints, highlighting the advantages of learning directly from the molecular structure. By tracing particular elements in the fingerprint back to the molecular substructures present in the molecule, some information about how the model makes decisions could also be obtained.

This ability for GCNNs with learnt molecular representations was further investigated by Yang et al.,^95^ in relation to a range of physicochemical property prediction tasks and resulted in another variant on the graph based approach, D-MPNN, in which information is placed on bonds rather than on atoms. Tests on the ESOL data set showed that this bond-centered approach outperformed an equivalent atom-centered network and also NNs trained on precomputed descriptors or fingerprints. Ryu et al. developed Bayesian GNNs for solubility prediction, with their test set encompassing 20% of the approximately 10000 compounds in the data sets. Despite the promise they saw in their results, as yet the work appears to have been published only as a preprint.^96^

Wiercioch and Kirchmair utilized a deep transformer model with a graph-based molecular representation to predict solubility for a set of approximately 130 compounds.^97^ Their study offers a valuable insight into the limitations that training set size places on such larger ML models. Even though they used transfer learning on a set of 6000 compounds with p*K*_a_ values to pretrain, their results still showed substantial dependence on data set size. When given over 1000 solubility values to train on, the transformer clearly outperformed a Random Forest benchmark. However, upon repeating that comparison with a reduced training set of 100 compounds, the transformer’s predictivity fell behind that of the Random Forest. As to molecular representations, Zheng et al.^98^ found that a graph-based approach required plentiful high-quality data to outperform traditional cheminformatics descriptors. These findings demonstrate the critical need for larger sets of verified high-quality solubility data, which will be essential to these larger models fulfilling their promise.

#### 2.2.3 Other NN Models

In chemoinformatics, molecules are often represented as text strings using the SMILES string format, or more recently SELFIES.^99^ This opens up the possibility of applying NN models designed for learning from text, such as transformers, to molecular property prediction. Francoeur and Koes developed SolTranNet for solubility prediction from SMILES strings based on a molecule attention transformer architecture.^100^ As a regression model, SolTranNet was found to perform worse than other deep learning approaches on the solubility challenge data set, however, it showed good accuracy at classifying insoluble compounds.

With the diversity of deep learning approaches and architectures available, it is not yet clear if particular architectures are more suited to deep learning and various comparison studies have been performed. Panapitiya et al. recently performed a thorough comparison of a range of different deep learning architectures.^101^ In contrast to earlier studies on GCNNs, they found that a NN trained on precomputed descriptors out-performed methods which learn directly from the chemical structure. Of these methods though the graph based approaches were the most promising. Further investigations are needed using consistent training and test sets to more conclusively determine the most promising approaches for predicting physicochemical properties and specifically solubility.

#### 2.2.4 Large Language Models

The rapid development of large language models (LLMs) in recent years has revolutionized how we access, interact with, and verify information. Those in the chemical sciences have already felt its far-reaching consequences, from integration into educational settings^102^ to its use in theoretical chemistry.^103–105^ While still a rapidly evolving method, large language models (LLMs) present a promising and intuitive method of solubility prediction. Since molecular property prediction is often limited by the expensive and time-consuming process of labeling data, LLM-based approaches typically employ zero-shot or few-shot scenarios where labeled data for use in learning is either limited or absent. There have been some interesting recent developments in chemistry-trained LLMs, for example in the QSAR/QSPR field.^106^ Zheng et al. recently presented a predictive LLM model pretrained on the academic literature, which they tested over a wide range of scientific problems, including both solubility and hydration free energy prediction, with very promising results.^107^ It will be interesting to observe future developments of this and similar models.

In 2023, Seidl et al.^103^ presented CLAMP (Contrastive Language-Assay-Molecule Pretraining), a new contrastive learning method which uses both textual and chemical data as input for activity prediction and which can adapt to new prediction tasks by ‘understanding’ the natural language information used to describe the task. CLAMP’s innovative modular architecture encodes language data and structure data separately before embedding the two data sources in a joint module. Seidl et al. evaluated their model’s effectiveness in three tests.^103^ First, a zero-shot transfer learning exercise to investigate whether the model could gain knowledge from textual data alone, with no chemical structure information; while Scientific Language Models (SLM) have shown ability to perform such tasks, previous prediction models fail.^108,109^ The CLAMP model was found to outperform all tested comparator methods, including SLM models, except for the frequency hitter model developed by Schimunek et al.,^110^ which does not make use of textual input but instead enriches the representation of the test molecule using information about similar molecules. The second test, representation learning, checked whether the molecular representations learned by CLAMP were transferrable across data sets. It was found that CLAMP performed better than other models on five of the eight data sets tested, and for those where it did not perform best, results were within one standard deviation of those of the best-performing model. The third test used CLAMP as a retrieval tool, enabling users to search a chemical database and find molecules ranked in priority for potential wet lab applications based on a given bioassay query. In this task, CLAMP outperformed the previously best-performing model, KV-PLM,^111^ by a multiple of 50 in its ability to rank highly those molecules which were active toward the query bioassay. The model’s accuracy in performing this task was based on the enrichment factor, which measures the accuracy of the top n results in a retrieval task. Given the success of these test results, CLAMP represents a foundational use of LLMs in molecular property prediction.

In 2024, Zhao et al.^104^ presented GIMLET (Graph Instruction based MolecuLe zEro-shoT learning), another tool for molecular property prediction which made use of a large language model-based approach. With their work, Zhao et al. sought to overcome two key issues with the CLAMP approach. The first was that the graph neural network on which CLAMP relies has only a limited ability to carry structural information. The second was caused by the inclusion of CLAMP’s additional joint embedding module. This additional module is difficult to train as “deep transformers have vanishing gradients in early layers”,^104,112^ which means that the gradients become so small that the network is unable to learn from the data in the additional module, and the module also incurs further cost and the need for additional parameters. To circumvent these issues, GIMLET unified graphical and textual data, encoding them without the need for an additional embedding module. The GIMLET method gave promising results in zero-shot scenarios and was applied to both classification and regression problems. While LLM-based approaches tend to struggle with regression problems due to difficulty in formatting numerical outputs, GIMLET generated correctly formatted numerical answers in 98% of its regression tasks. The GIMLET method was used for zero-shot aqueous solubility prediction for a set of molecules, using the following textual instruction:

“Solubility (logS) can be approximated by negative LogP −0.01 * (MPt - 25) + 0.5. Can you approximate the logS of this molecule by its negative logP and MPt?”

GIMLET therefore used textual input to provide the model with the General Solubility Equation. Zhao et al. noted a “strong correlation” between predicted and experimental results for the zero shot prediction, with a RMSE of 1.132, compared to 1.331 for a supervised graph convolution network and 1.253 for a supervised graph attention network approach. GIMLET was outperformed by the supervised Graphormer method,^113^ with an RMSE value of 0.901, or of 0.804 when using a pretrained data set. However, when GIMLET was used to make few shot solubility predictions, results outperformed the supervised general intelligence network. The GIMLET model represents a significant step forward in solubility prediction, offering results which outperform other several supervised methods, and which allows for straightforward user input in natural language.

However, Liu et al. later noted that CLAMP and GIMLET lacked the extensive generalization that LLMs possess when used in natural language processing, such as the GPT softwares.^105,114^ Liu et al. therefore presented MolecularGPT,^105^ a LLM which can be generalized to a variety of molecular property prediction tasks in few shot and zero shot scenarios. Their work sought to unify data corresponding to molecules of different sizes, densities and chemical space into a single consistent format. MolecularGPT uses SMILES codes as a method of converting graph information to string information.^115^ For predicting ESOL water solubility on the same data set as was used to test GIMLET, Molecular GPT predictions gave a RMSE of 1.471, higher than the 1.132 of GIMLET.^104^ However, MolecularGPT was more consistent in its answers than GIMLET when the type of instruction is changed; MolecularGPT’s predictions had a standard deviation of 0.007 with respect to its RMSE across five different instruction types, while GIMLET had a standard deviation of 0.020.^104,105^

Both MolecularGPT and GIMLET represent the cutting edge of natural language processing in the prediction of solubility, as well as having the power of generalized molecular property prediction.

### 2.3 Empirical “Physics-Inspired” Models

Alongside empirical, data-driven methods for solubility prediction, there are also a number of semiempirical methods which use theoretical, physics-based arguments to construct simple relations between solubility and other calculated or experimentally determined parameters.

#### 2.3.1 General Solubility Equation

In 1980, Yalkowsky and Valani proposed the first version of the General Solubilty Equation (GSE).^116^ Subsequently revised by Jain and Yalkowsky,^82^ this equation relates solubility, log *S*, to the octanol–water partition coefficient, log *P*, and melting point (MP), *T*_*m*_,

12 $$\log S=0.5-\log P-0.01\left(T_{m}-25\right)$$

where temperature is measured in degrees Celsius. For liquid solubility, *T*_*m*_ is set to 25 °C so that the MP term is zero. The equation describes the intrinsic solubility of organic compounds, although modifications to extend it to weak electrolytes have also been described.^117^

The form of the GSE can be understood by considering the fusion cycle. The melting point term is associated with the free energy of fusion, which must be overcome to melt the solid and produce a supercooled liquid solute phase. This represents the ideal solubility, in which the enthalpy of solute–solvent mixing is zero. The log *P* term accounts for the solvation of the solute as the supercooled melt mixes with the aqueous phase. log *P* has been shown to correlate approximately linearly with solubility,^118^ a relationship which also explains the prevalence of log *P* descriptors in QSPR models for solubility prediction. The GSE contains no trainable parameters and the values of the coefficients and intercept term have not been obtained by fitting to experimental data. However, fitting equations of the same general form to experimental solubility data sets has resulted in similar parameters for the coefficients and intercept and only slight improvements in prediction accuracy.^117,119,120^

Solubility predictions using the GSE can be made with experimental log *P* and MP data if available, but calculated log *P* descriptors, such as the atom contribution approach of Wildman and Crippen^81^ can also be used without too great a loss of accuracy (for example the RMSD increased from 0.52 to 0.62 on the Jorgensen and Duffy data set).^119^ Replacing the experimentally determined MP with a predicted MP value is more difficult due to the limited performance of current MP prediction models with RMSDs of around 31 – 40 °C.^121^ However, McDonagh et al. demonstrated that useful qualitative predictions can be made using a RF model to predict the MP,^122^ especially considering the relatively low weighting of the MP term in the GSE.

Removing the reliance on experimental data enables the use of the GSE to be extended to unsynthesised compounds. Taking inspiration from the form of the GSE, Hill and Young proposed the Solubility Forecast Index (SFI), defined as SFI = *c* log *D*_pH 7.4_ + *N*_aromatic rings_, to estimate the level of solubility for drug-like compounds, with the general guideline that compounds with an SFI < 5 are likely to have reasonable solubility and other physicochemical properties.^123^

Jain and Yalkowsky also developed an alternative model to the GSE called SCRATCH,^124^ which uses no experimental parameters. Instead SCRATCH combines the aqueous activity coefficient, calculated from the AQUAFAC group contribution approach,^125^ with the predicted MP, based on the enthalpy and entropy of melting, calculated using a group contribution approach and descriptor based equation^126^ respectively. This showed only slightly reduced performance compared to the GSE.

The GSE has been tested on a number of common benchmark test sets, with RMSDs of 1.23 (first solubility challenge), 1.10 and 1.24 (second solubility challenge tight and loose test sets respectively), using experimental MPs.^6,127^ The GSE works well on small compounds, but is less accurate on the larger compounds found in pharmaceuticals or pesticides. This is in part due to the assumption of Walden’s rule, which states that the entropy of melting is constant, and which breaks down for larger and more flexible compounds.

To address this limitation, Avdeef and Kansy extended the GSE by including two additional parameters to account for molecular flexibility and hydrogen bonding, which modify the log *P* and MP term coefficients and intercept in the original GSE.^128^ The flexibility was accounted for using the Kier index,^129^ following the suggestion from Caron et al.,^130^ while hydrogen bonding was included using the Abraham H-bond acceptor parameter, *B*. Unlike the original GSE, the contributions of these additional terms were fit to experimental data using a large data set of intrinsic solubility data.^127^ The resulting “Flexible Acceptor” GSE showed considerable improvement on larger more flexible, compounds, specifically “beyond rule of 5” (bRo5) compounds which break Lipinski’s rules of 5 (Ro5)^8^ compared to the original GSE (RMSD reduced from 3.0 to 1.1), without sacrificing much of the simplicity of the original GSE equation. A subsequent study found that this “Flexible Acceptor” GSE also outperformed the original GSE and an RF model on a test set comprising newly approved drug compounds covering both Ro5 and bRo5 chemical space.^131^

#### 2.3.2 Hansen Solubility Parameters

The GSE is limited to aqueous solubilities, due to the use of the octanol–water partition coefficient to account for the solute–solvent interactions. However, other approaches have been developed to estimate solubility in different solvents.

In 1950, Hildebrand and Scott introduced the solubility parameter, δ^2^, defined as the interaction energy density for a given compound,^132^ with the idea that solutes and solvents with similar values of δ^2^ should be mutually favorable for solubility. The rationale was that molecules will be soluble if the strength of solvent–solvent and solute–solute interactions is similar to the strength of solvent–solute interactions, so that the enthalpic penalty to mixing is minimized. This idea is often expressed by the rule of thumb that “like dissolves like”. The approach was developed further by Hansen,^133^ by splitting the total energy density into three Hansen Solubility Parameters (HSP) to separately describe the contributions of the dispersion, δ_*D*_^2^, polarity, δ_*P*_^2^, and hydrogen bonding, δ_*H*_^2^, to the overall energy density, where δ^2^ = δ_*D*_^2^ + δ_*P*_^2^ + δ_*H*_^2^.

The HSP define the coordinate axes of a 3D space in which solvents and solutes can be arranged. The distance between solute *i*, and solvent *j*, within this HSP or Hansen space is defined as

$$R_{a}=\sqrt{4{\left(\delta_{D,i}-\delta_{D,j}\right)}^{2}+{\left(\delta_{P,i}-\delta_{P,j}\right)}^{2}+{\left(\delta_{H,i}-\delta_{H,j}\right)}^{2}}$$

­(note that the dispersion term is scaled by a factor of 4).^134^ Solutes are soluble in solvents which are close in HSP space, and the boundary between soluble and insoluble mixtures is represented by the Hanson sphere, which has radius *R*_*o*_. The location of solvents relative to the edge of the Hansen sphere can be described by the Relative Energy Difference (RED), RED = *R*_*a*_/*R*_*o*_, where RED < 1 indicates a solvent is inside the Hanson sphere and the solute is likely to be soluble.

Originally the HSP approach was applied to find suitable solvents for polymers, however, it has been extended to look at solubility in a much wider range of systems, including ionic liquids^135^ and lipid bilayers for drug delivery.^136^ Interest in HSP methods has also increased with the growing focus on green chemistry and in particular the search for alternative solvents with a lower environmental impact.^137^ Modifications to the form of the HSP parameters have also been suggested to better capture the behavior of small molecules and to extrapolate results to different temperatures.^138^

HSP values for new solutes can be found from experiment, however a variety of methods have also been used to predict HSP, many based on group contribution approaches. For example, Stefanis et al. used a combination of UNIFAC functional groups, supplemented with second-order groups to account for the effect of conjugation.^139^ Alternatively, Mathieu suggested a 3 parameter equation which takes the molar volume and a GC based estimate of the molar refractivity, and is fit to experimental *δ*_*D*_ data.^140^ This was combined with a fragment based model to predict *δ*_*H*_ and *δ*_*P*_, based on a more limited subset of fragments which significantly contribute to molecular polarity and hydrogen bonding. The use of the molar refractivity proved to be an improvement for *δ*_*D*_ prediction, however, the more extensive training set of Stefanis et al. gave better predictions for *δ*_*P*_.

HSP values have also be calculated using information from other physics-based methods. Jarvas et al., used σ-moments calculated from COSMO-RS as input to a NN with a single hidden layer.^141^ They found that they were able to predict HSP for a diverse set of compounds including salts and ionic liquids and that a NN was an appropriate model to capture the nonlinear relationship between σ-moments and HSP. More recently, Sanchez-Lengeling et al. have developed a workflow which combines various techniques including simulation, DFT and COSMO-RS calculations to obtain descriptors to train a Gaussian Process model.^142^ This model, named gpHSP, provides both an estimate of the three HSP parameters and the associated prediction uncertainties and has a predictive performance approaching the experimental error in measured HSP values.

## 3 Physics-Based Methods

Physics-based methods of solubility prediction are largely based on first-principles, so do not require parametrization against experimental solubility data in the way that data-driven methods do, and are capable of providing chemical insight. Over the last few decades, many different physics-based methods for solubility prediction have been developed. These methods fall broadly into two groups, which will be reviewed in detail: (i) direct methods, which compute solubility directly from simulation; and (ii) indirect methods, which calculate thermodynamic parameters that are then combined to obtain solubility, often based on a thermodynamic cycle.

### 3.1 Direct Calculation of Solubility

Direct solubility calculations rely on simulating the system of interest. Several distinct methodologies exist, including the direct coexistence method, in which solid-phase and solution-phase solute molecules are equilibrated in a molecular dynamics simulation;^143,144^ the chemical potentials route, which leverages the equivalence of the solid-phase and solution-phase chemical potentials at the solubility limit;^145^ the density of states approach, which offers a distinctly different methodology;^146,147^ and the Einstein crystal method, which uses thermodynamic integration from a hypothetical crystal.^148^ These methods have been used to predict aqueous and nonaqueous solubilities of simple salts and small organic molecules. However, they are often limited by high computational cost and the ability of the chosen force field to represent a range of solute molecules in both solid and solution phases in a single simulation. Therefore, the following discussion tracks efforts to improve computational efficiency and provide accurate, generalizable results.

#### 3.1.1 Direct Coexistence

Commonly known as the ”brute force” approach, direct coexistence uses molecular dynamics simulations to simulate two adjacent periodic cells, one populated with the solid-phase solute, the other with the solution-phase solute in a selected solvent, at a particular temperature and pressure.^143,144^ Simultaneous dissolution and crystallization processes are then simulated until the overall system reaches equilibrium. The solute’s equilibrium concentration, which represents its solubility limit under the given conditions, is then determined by simply counting the number of solute and solvent molecules in the solution-phase cell. This method benefits from theoretical simplicity, with a relatively straightforward setup and execution. However, the direct coexistence method is limited by high computational cost, which has two root causes.

The first is that, in order for the solute and solvent molecules to be counted, the molecular dynamics simulation requires an explicit solvation model. This results in high computational costs meaning that, in practice, the molecular dynamics simulations are limited to small systems. This hinders the precision of the method’s predictions, since systems with larger atom counts allow for more precise calculation of the equilibrium concentration. Furthermore, the need for an explicit solvation model is an intrinsic limitation of the method, since less costly representations of the solvent such as a polarizable continuum model, in which the solvent is represented by a single, homogeneous, polarizable field, would not allow for atom counting. The method does, however, benefit from phenomena such as solvation shells being accounted for by the explicit solvation model. The second root cause of the high computational cost is the time taken for the system to equilibrate, which in some cases can be up to several microseconds.^143^ Since molecular dynamics simulations typically employ timesteps on the order of femtoseconds, on the order of a billion simulation steps are required for the system to equilibrate.

Another limitaton of the direct coexistence method is that in order to simulate the dissolution process of a query compound, the crystal structure of the compound must be known.^149,150^ Later in this review, we discuss the feasibility of instead basing the calculation on a model structure derived from Crystal Structure Prediction (CSP). However, the method has so far been applied primarily to simple alkali halides, the structures of which are well documented.

Manzanilla-Granados et al. used the direct coexistence method to predict aqueous sodium chloride solubility, using the JC-TIP4P-Ew models for water, and Joung and Cheatham’s parameters for the sodium chloride species.^143,151,152^ The group sought to compare four distinct initial systems within the simulation and monitor the impact on computational cost and prediction accuracy. The first system comprised a sodium chloride solution between two solid-phase sodium chloride plates; the second an aqueous sodium chloride solution with ions distributed randomly, and no solid-phase sodium chloride; the third a block of solid-phase sodium chloride submerged in pure water; and the fourth a block of solid-phase sodium chloride submerged in a supersaturated aqueous sodium chloride solution. It was found that the solubility prediction, which averaged 4.2(3) mol kg^–1^ across the four setups, did not significantly change between setups, within simulation error. It was, however, observed that the fourth setup equilibrated much faster than the other three setups, affording a more tractable computational cost without hindering the prediction accuracy. However, it is not clear if these results are transferrable to other systems. The group also tested the first and fourth setups using the JC-SPC/E water model rather than the JC-TIP4P-Ew model,^151,153^ which gave an average prediction of 5.9(3) mol kg^–1^, highlighting the force field-dependence of solubility predictions. Both of these predictions are in reasonable agreement with an experimental value of 6.15 mol kg^–1^ (298.15 K, 1 bar).

Later, Kolafa applied the polarizable AH/BK3 model for both the ions and the water molecules,^154–156^ to predict the aqueous solubility of sodium chloride. The AH/BK3 model represents ionic charges using Gaussian charge distributions which are connected to their gas-phase positions by harmonic springs. The mechanical equilibrium between the electrostatic forces and the spring forces determines the polarizability of the ion. The AH/BK ion model also features exponential repulsion and the r^–6^ attraction functions (where *r* is the interatomic/interionic distance). The ion and water force fields are ‘transferable’, meaning that the potential of ion–ion, ion-dipole and dipole–dipole interactions can be described as a simple combination of the two interacting potentials. Kolafa found that this method produced a reliable, albeit inaccurate, result of 0.56(15) mol kg^–1^, which is a less accurate prediction that those given by Manzanilla-Granados et al.^143^ In the same paper, however, Kolafa modified the AH/BK3 force field to create the MAH/BK3 model, retained some key features such as the Gaussian charge distribution, the charge-on-spring polarizability, and the repulsion and attraction functions, but refitting the alkali halide crystal’s thermomechanical parameters. This refitting rectified an issue of the simulated sodium chloride crystal being too stable, which had resulted in the underestimation of the crystal’s solubility. The MAH/BK3 model gave a more accurate solubility prediction of 3.7(3) mol kg^–1^. Kolafa also tested the JC-SPC/E ion model, previously used by Manzanilla-Granados et al.,^143^ which yielded a prediction of 3.6(3) mol kg^–1^, in reasonable agreement with that of the MAH/BK3 model.^144^ Unlike the MAH/BK3 and AH/BK3 models, the JC-SPC/E model is a nonpolarizable force field which represents ions using elementary point charges rather than Gaussian charges.

Perhaps the most accurate results using the direct coexistence method come from Yagasaki et al.,^157^ who investigated pairing alkali halide models with a variety of different water models and comparing the results. The group developed Lennard-Jones parameters for sodium (Na^+^), potassium (K^+^) and chloride (Cl^–^) ions and predicted the aqueous solubility of sodium chloride and potassium chloride using three water models: SPC/E,^153^ TIP3P^158^ and TIP4*P*/2005.^159^ The sodium chloride model performed best when paired with the TIP3P model, though only by a small margin, giving a prediction of 6.1(3) mol kg^–1^. The potassium chloride model gave a consistent result of about 4.7 mol kg^–1^ with all three water models, which agrees with the experimental value of 4.76 mol kg^–1^. Despite yielding reasonably accurate results, the Yagasaki et al. study is limited by several factors. Since the simulations were conducted at ambient conditions, it is unclear if the models can capture the temperature dependence of solubility. Further, it is unclear if the Lennard-Jones parameters developed for Na^+^, K^+^ and Cl^–^ ions are transferable to other alkali metal and halide ions, providing opportunities for further research.

The direct coexistence method offers a theoretically straightforward route to solubility prediction. Its notoriously long equlibration times have been tackled by Manzanilla-Granados et al.,^143^ however it is unclear whether their results are generalizable to other systems. While recent studies have yielded promising results,^157^ these results were obtained from specially developed Lennard-Jones parameters which may not transfer to other alkali halides. Perhaps most importantly, studies using the direct coexistence approach have been limited to simple alkali halides. To predict the solubility of organic molecules, more sophisticated approaches are required.

#### 3.1.2 Chemical Potentials Route

The chemical potential route (CPR) is a method of solubility prediction which leverages the fact that, under given temperature and pressure conditions, the solubility limit occurs when the chemical potentials of the solid-phase solute and solution-phase solute are equal.^145^ The chemical potential of the solid-phase solute is defined as the Gibbs free energy per molecule, while the chemical potential of the solution-phase solute, which varies with solution concentration, is defined as the Gibbs free energy change resulting from the addition of one solute molecule into the solution. CPR methods typically use Monte Carlo (MC) and molecular dynamics (MD) simulations to calculate the chemical potential of the solid-phase solute, and determine the chemical potential of the solution-phase solute as a function of the solution concentration. This enables the solubility limit to be predicted by satisfying the equality of the two chemical potentials, where the corresponding solution concentration represents the solubility limit (see Figure 1). While such methods have been used to predict aqueous solubilities of organic compounds,^160^ much of the development of the method is concerned with predicting solubilities for simple alkali halides.^145^

**Figure 1:**
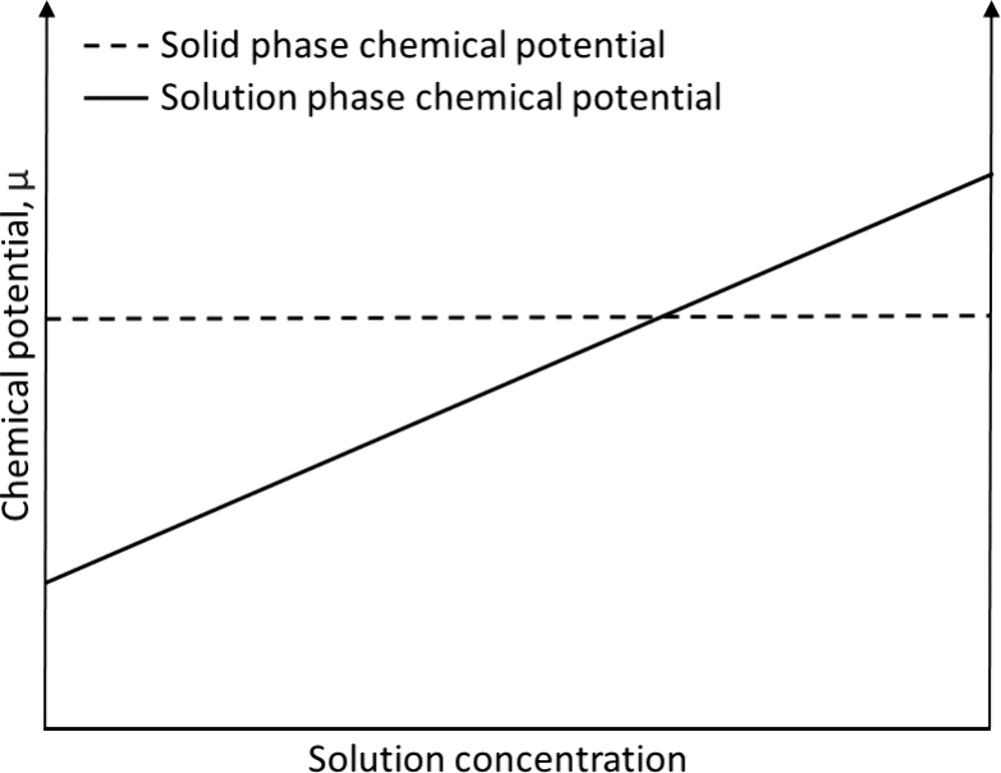
Illustrative example of the chemical potentials approach to solubility calculation. The solubility limit can be determined by finding the point at which the chemical potentials of the solid-phase and solution-phase solutes are equal.

In 2015, the CPR method was used with four different ion models, each paired with the SPC/E water model,^153^ to predict the aqueous solubility of NaCl at ambient conditions, revealing significant issues.^161^ The four ion models were: KBFF (a nonpolarizable force field);^162^ RDVH (a force field which represents ions using Lennard-Jones potentials with superimposed elementary point charges);^163^ JC-SPC/E (a nonpolarizable force field which represents ions as elementary point charges, as described in Section 3.1.1.);^144^ and SD (a polarizable force field which represents ions using Lennard-Jones potentials).^164^ The best prediction was made by the RDVH model (5.69(7) mol kg^–1^), followed by the JC-SPC/E model (3.71(4) mol kg^–1^), the KBFF model (0.88(2) mol kg^–1^) and the SD model (0.63(1) mol kg^–1^). The models were then tested for their transferability to elevated temperatures: the JC-SPC/E and RDVH models predicted a decrease in solubility with increasing temperature, while the KBFF and SD models predicted little influence of temperature on solubility. According to the Gibbs function, the incorrect temperature dependence of the JC SPC/E and RDVH models suggests that the dissolution process had a negative entropy change. This indicates that the entropy of the modeled crystal was too high, the entropy of the modeled solution was too low, or both, which may have been caused by overfitting the model. In 2022, the solubility of NaCl in a water–methanol mixture was calculated for varying solvent ratios ranging from pure water to pure methanol. Despite not giving the best predictions in their 2015 model, the group used the Joung-Cheatham model for the salt, the SPC/E model for the water, and the OPLS-AA model for the methanol, and achieved “surprisingly good” agreement with experimental data.^165^ The solution chemical potential was calculated by insertion of a single ion into the solution, with a neutralizing background, and this was compared with the solid-state potential calculated from the group’s 2015 paper.^161^

In 2020, Dočkal et al. reparameterised the existing AH/BK3 and MAH/BK3 models (which were previously discussed with regards to their use in the direct coexistence method) and applied the resultant reparameterised model (RM) to a CPR method.^166^ The RM gave a good aqueous NaCl solubility prediction of 7.0(4) mol kg^–1^ (ambient conditions). The RM was tested at elevated temperatures, giving values of 6.7(6) mol kg^–1^ (373.15 K, 1 bar) and 6.8(6) mol kg^–1^ (473.15 K, 15.5 bar). While these predictions could reflect the temperature dependence of solubility, within one standard deviation, their ability to do so was not conclusively proven. Dočkal et al. pointed out that their reparameterisation process is generalizable to other simple alkali halide salts, although this is yet to be tested. The group also noted that the inaccuracies in the predictions of their model were likely due to the ion–water interactions still being modeled by the AH/BK3 model. Reparameterising these interactions could yield even more accurate results, but this is yet to be done, highlighting another opportunity for future work. There are several other opportunities for future work. The RM is currently more attractive than other modern models such as the Madrid model,^167^ as it does not improve solubility predictions at the expense of predictions of other properties. However, the temperature dependence of the Madrid model has not yet been tested; the ability to capture such trends could make it more advantageous than the RM, providing an important topic for future work. Further, several modern polarizable water models, such as E3B and HBP, have not had compatible ion models designed for them so their solubility prediction has not yet been tested.^168,169^

While the chemical potentials route has mostly been reserved for simple alkali halides, it has been applied to alkaline earth metal halides..^170^ This presents a challenge due to the slow dynamics of such systems, and the fact that alkaline earth metal halides are hydrated in the solid phase but not in the liquid phase. The group used three force fields for the molecular dynamics simulations: Mamatkulov et al.,^171^ DRVH,^172^ and ECCR2,^173^ with ECCR2 giving the most accurate predictions of solubility.

A common problem with the chemical potentials route to solubility prediction is the need to convert the modeling of the solid-phase solute, which has bound degrees of freedom, to the solution-phase solute, which has additional translational and rotational degrees of freedom. Khanna et al. took first steps to deal with this problem by positing a decoupled approach to calculate chemical potentials at a solid–liquid phase equilibrium which starts from two independent reference systems.^174^ The group adopted the Frenkel-Ladd method for obtaining the solid-state chemical potential,^148,175,176^ in which an Einstein crystal is used as a reference system, its chemical potential is calculated, and it is converted into a fully interacting crystal in a stepwise fashion. The change in chemical potential of each of these steps can be calculated, hence the chemical potential of the fully interacting molecular crystal can be found. The Frenkel-Ladd method is further discussed in Section 3.1.4. However, to avoid the solid-to-liquid transition, the group employed an independent reference system for the liquid-phase compounds, known as the *centroid*. The goal of the centroid approach to calculating liquid-phase chemical potentials is to give the reference system the same number and types of degrees of freedom as are present in the fully interacting molecule, as the closer the reference system is to the real system, the easier it is to transform the reference system into the fully interacting system; difficulties often arise, for example, when ideal gases are used as reference systems as they possess fewer degrees of freedom than real gases. The centroid is composed of all of the atoms present in the real system, each bound by a spring to the collective center of mass. The chemical potential of the centroid can be computed either analytically or through simulation, and it is then transformed into the fully interacting molecule in a series of steps, first by bonding the atoms together as they are in the real molecule, then by gradually turning on correct angles, dihedrals and Lennard-Jones interactions, and finally by turning on Coulombic interactions (see Figure 2).

**Figure 2:**
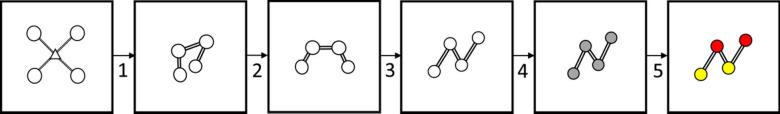
Stepwise conversion of a centroid to a fully interacting molecule. 1. The centroid system transforms to bonded atoms; 2. bond angles are turned on; 3. dihedrals are turned on; 4. Lennard-Jones interactions are turned on; 5. Coulombic interactions are turned on. Reproduced with permission from Reference 174. Copyright 2020 American Institute of Physics (AIP) Publishing.

Each of these steps has an associated chemical potential change, which can be added to the chemical potential of the reference system, hence affording the chemical potential of the fully interacting molecule. While the group originally used this method to compute solid–liquid phase equilibria, it was later extended to solubility calculation in 2023 to predict the aqueous solubilities of naphthalene and β-succinic acid.^160^ These calculations required additional solvation free energy calculations at varying concentrations. The predicted solubility of naphthalene as a mole fraction of 3.66(0.2) × 10^–6^ is in reasonable agreement with the experimental value of 4.4 × 10^–6^, and the calculations display a linear increase in solubility with temperature.^160^ However, the predicted solubilities across a temperature range of between 300 and 350 K for succinic acid were around three times larger than experimental values. Given the rigidity of naphthalene and the relative flexibility of succinic acid, these results indicate that the centroid method performs well for rigid compounds but poorly for more flexible compounds. However, the standard error of the solubility calculations overall remains below 10%, making the centroid method of the chemical potentials approach a promising candidate for future development. Khanna et al. concluded that overcoming inaccuracies in the parts of the force field modeling solute–solvent and solvent–solvent interactions would be particularly beneficial.

Another significant step forward in the chemical potentials route came recently from Reinhardt et al., who sought to provide a streamlined molecular dynamics methodology.^177^ To model a solution’s chemical potential as a function of concentration, the group used a Debye crystal of a known energy, in which all pairs of atoms were connected by harmonic springs, as a starting point for thermodynamic integration, which was used to switch off the harmonic Debye interactions and switch on the potential, creating an interacting crystal. Then, a Debye solute molecule, in which all atom pairs were connected by harmonic springs and the center of mass and rotational movement were constrained, underwent Hamiltonian thermodynamic integration followed by free-energy perturbation to convert it into a fully interacting molecule. Finally, free-energy perturbation was used to add a softly interacting solute molecule into a very dilute solution of concentration *C*_0_, the potential of which was gradually switched on to become a fully interacting solute molecule in solution. The solvation free energy was calculated as the difference in free energy between the solute molecule fully interacting in dilute solution and the same molecule in the gas phase. The *S* ^0^ method was then used to calculate the chemical potential, *μ* ^*sol*^, as a function of solution concentration, *c*. The *S* ^0^ method is based on the thermodynamic relationship between the changes in particle numbers and derivatives of the chemical potentials with respect to molar concentration,

13 $$\begin{array}{l} \mu^{sol}\left(c\right)=\mu^{sol}\left(c_{0}\right)+k_{B}T\ln\left(\frac{c}{c_{0}}\right)+k_{B}T\int_{\ln c_{0}}^{\ln c}d\ln\left(c\right)\left[\frac{1}{S_{M-M}^{0}-S_{M-S}^{0}\sqrt{\frac{c}{c_{S}}}}-1\right] \end{array}$$

where the *M* and *S* subscripts represent solute and solvent molecules, respectively; *S*_*M*–*M*_^0^ is the static structure factor in the *k* → 0 limit between solute molecules, and *S*_*M*–*M*_^0^ is the same between solute and solvent molecules. This methodology was used to predict solubilities of sodium chloride in water, urea polymorphs in water at a range of temperatures, and paracetamol in water and in ethanol. The method tended to underestimate solubilities, for example giving a value of (3.5 ± 0.5) × 10^–8^ mol kg^–1^ for aqueous paracetamol at 20 °C, compared to the experimental value of 0.0845 mol kg^–1^ under the same conditions.^178^ This shortcoming is likely due to the breakdown of the harmonic potential approximation, which is key to the Debye crystal, at elevated temperatures. However, the increased computational efficiency of this method, and its ability to reasonably accurately predict the solubility of simple alkali halides, makes it a promising candidate for future work in solubility prediction for organic molecules.

The chemical potentials route offers a reliable method of solubility prediction which leverages the equivalence between the solid phase and solution phase solute at the solubility limit. Such methods are computationally less intensive than direct coexistence methods, especially with recent steps forward,^177^ and can provide accurate solubility predictions for simple alkali halides. While they tend to give predictions of varying quality for small organic molecules,^160^ predictions remain heavily dependent on the force field used. Such methods can also become computationally cumbersome when calculating solubilities under a wide set of temperature and pressure conditions. However, a different approach, using the system’s density of states, is able to straightforwardly access solubility predictions at a range of temperatures and pressures.

#### 3.1.3 Thermodynamic Approaches (Density of States)

In response to the abundance of simulation-intense solubility prediction methods such as those discussed above, Boothroyd et al. proposed an efficient way of accessing solubility predictions across a range of temperatures and pressures using only a small number of simulations.^146,147^ Their approach involved calculating a solute–solvent system’s density of states, which is the number of configurations that that system may occupy at a given energy,^179^ in order to identify the point of phase coexistence between the solid phase and solution phase solute, thus accessing the solubility. By applying well-established density of states calculations, which have historically been limited to single-component systems,^180,181^ to multicomponent systems, Boothroyd et al. accessed solubility predictions for simple salts and organic molecules.

For a single-component system, the point of equilibrium can be determined from the density of states by first considering the isothermal–isobaric (NpT) partition function in which distinct microstates may be degenerate. This is expressed as

14 $$Q\left(N,p,T\right)=\underset{E}{\sum }\underset{V}{\sum }\Omega \left(V,E\right)\exp^{-\beta \left(E+pV\right)}$$

where Ω­(*V*, *E*) is the system’s density of states, *E* and *V* are the system’s energy and volume, respectively, and the summation is over all energy levels. Knowing the system’s density of states allows for scanning of the corresponding probability distribution,

15 $$P\left(E,V\right)=\frac{1}{Q\left(N,p,T\right)}\exp^{\ln\Omega \left(V,E\right)-\beta \left(E+pV\right)}$$

across either temperature or pressure while keeping the other constant, affording access to coexistence conditions. For multicomponent systems containing species *i* and *j*, where the population of species *i* (*N*_*i*_) is allowed to change and the population of species *j* (*N*_*j*_) is constant, the partition function is given by

16 $$Q{\left(\mu_{i},p,T\right)}_{N_{j}}=\underset{E}{\sum }\underset{V}{\sum }\underset{N_{i}}{\sum }\Omega {\left(N_{i},V,E\right)}_{N_{j}}\exp^{-\beta \left(E+pV-\mu_{i}N_{i}\right)}$$

where *μ*_*i*_ is the chemical potential of species *i*. Again, if the density of states, here Ω­(*N*_*i*_,*V*,*E*)*N*_*j*_, is known, the corresponding probability distribution,

17 $$P{\left(N_{i},E,V\right)}_{N_{j}}=\frac{1}{Q{\left(\mu_{i},p,T\right)}_{N_{j}}}\exp^{\ln\Omega {\left(N_{i},V,E\right)}_{N_{j}}-\beta \left(E+pV-\mu_{i}N_{i}\right)}$$

can be used to identify the coexistence point at which the solid phase and solution phase component *i* are in equilibrium. This probability distribution as a function of *N*_*i*_ displays two peaks of equal area, representing the two coexistence states: one at *x*_*i*_ = 1, representing a solid component *i*, and the other at some lower mole fraction, 0 ≤ x_*i*_ < 1, which corresponds to the saturated solution, i.e., the solubility limit (see Figure 3). Therefore, the mole fraction at which the peak corresponding to the saturated solution occurs is the solubility limit.

**Figure 3:**
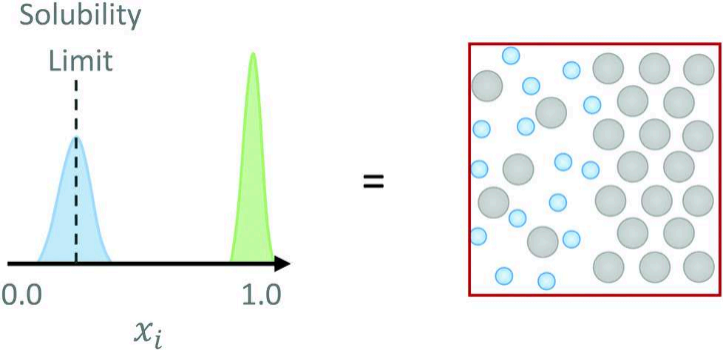
Illustrative example of a probability distribution for a system of solute (gray particles) and solvent (blue particles) as a function of solute fraction (*χ*_*i*_). At the solubility limit, the solute particles will have an equal probability of being in both the solid phase (the green peak at *χ*_*i*_ = 1.0) and the solution phase (the blue peak). The location (mole fraction) of the solution phase peak is the solubility limit. Reproduced with permission of the Royal Society of Chemistry from ref 146. Available under a Creative Commons Attribution 3.0 Unported License. Copyright 2018 Royal Society of Chemistry.

This method was first used to predict the aqueous solubility of sodium chloride at ambient conditions; the prediction of 3.77(5) mol kg^–1^ agreed with values predicted by previously described methods and is comparable to the experimental value of 6.15 mol kg^–1^.^146^ The key advantage of this method is that, since the density of states is independent of temperature and pressure, a system’s solubility limit can theoretically be predicted at a range of temperatures and pressures from only one density of states calculation. This method is therefore efficient in solubility prediction across a variety of conditions. However, in order to plot an accurate probability distribution for solubility prediction, a large number of free energy calculations are required across a wide range of possible discrete mole fractions. Further, although these numerous calculations are necessary, many of them will not be directly used for solubility prediction, as only those corresponding to the relevant peak in the probability distribution directly offer useful information about the solubility limit. However, the group later tackled these inefficiencies.

A subsequent alteration to the method used the density of states to calculate the system’s free energy of solution at a given concentration, rather than to calculate coexistence conditions.^147^ Performing several free energy calculations across a range of concentrations allowed for a free energy of solution versus concentration function to be fitted; the system’s chemical potential was then accessible as the first derivative of this function. Leveraging the fact that the solubility limit occurs at the equivalence of the solid-phase and solution-phase chemical potentials, the intersection of this solution-phase chemical potential function with that of the solid-phase chemical potential provided access to the solubility.

This modification was a step forward in efficiency. While the revised method required several free energy calculations in order to produce an accurate free energy function, far fewer calculations were required than in the original method. Moreover, all of the calculations in the modified method were pertinent to the final prediction, making it more information-efficient. The modified approach also retained advantages associated with the original density of states method, specifically the ability to calculate solubilities across a range of temperature and pressure conditions with only one density of states calculation. This method was used to determine the solubility of urea both in water and methanol. The urea and methanol molecules were modeled using the General AMBER Force Field and the water molecules were modeled using the TIP3P force field.^158,182^ The solubility prediction in methanol, 0.85(3) mol kg^–1^, was somewhat comparable to the experimental value of 4.01 mol kg^–1^. However, the method failed badly for the aqueous solubility of urea, giving a prediction of 0.46 mol kg^–1^, which is about 45 times lower than the experimental value of 20.15 mol kg^–1^. Despite this shortcoming, the method captured the correct temperature–dependence of solubility for both solvents, and gave accurate relative solubilities in both cases. According to Boothroyd and Anwar, the failure of the absolute solubility predictions and success of the relative solubility predictions suggests that “something systematic may be missing from the force fields”.^147^

The density of states approach, particularly the updated methodology,^147^ provides a method of solubility prediction for organic molecules in both aqueous and nonaqueous solvents which is far more computationally efficient that the direct coexistence and chemical potential routes. The method allows solubility calculation across a range of temperatures and pressures from only one density of states calculation and, while absolute solubility predictions have so far been poor, offers reasonably accurate relative solubility predictions. Like many physics-based methods, the density of states approach may be improved by better-parametrized force fields. However, no further improvements to the method have been reported since.

#### 3.1.4 Einstein Crystal Method

An atomic Einstein crystal is a hypothetical crystal in which each atom is bound to its lattice position by a harmonic spring; all springs in the crystal oscillate at the same frequency^175^ Since the force constant of the springs is known, the Helmholtz free energy of the crystal can be calculated, and hence the chemical potential of the solid can be found analytically. For molecular Einstein crystals, several springs are required per molecule: one central spring binds the molecule’s center of mass to its lattice position, while several more hold the molecule in the same orientation as all other molecules in the crystal.^148^ The chemical potential of the molecular Einstein crystal can similarly be found analytically.

The Einstein Crystal method, developed by Frenkel and Ladd in 1984, used Monte Carlo simulations to compute the free energy of the solute crystal of interest by thermodynamic integration to the real crystal from an atomic Einstein crystal of the same structure.^175^ The extended Einstein crystal method (EECM), proposed in 2017, is a straightforward extension of the original method and was used to successfully predict the aqueous solubility of naphthalene.^148^ To compute the free energy of the naphthalene crystal, MD simulations were employed to calculate the free energy changes associated with a series of reversible steps which transformed the reference molecular Einstein crystal (the free energy of which was known) into the real naphthalene crystal, hence finding the free energy (and subsequently the chemical potential) of the real naphthalene crystal. The chemical potential of the aqueous naphthalene solution was then determined using an MD simulation to insert a naphthalene molecule into a cavity in simple point-charge (SPC) water, then shrink the cavity away, leaving only the naphthalene molecule in the solution. The predicted solubility value of 4.74 × 10^–6^ mol kg^–1^ compared well with the experimental value of 4.40 × 10^–6^ mol kg^–1^.

In 2019, the EECM was employed by a different group to predict the solubility of acetaminophen (paracetamol) in ethanol.^176^ The predicted solubility of 0.085(14) mol L^–1^ compared well with the mean experimental value of 0.059(4) mol L^–1^. Acetaminophen was selected as a test solute due to its pharmaceutical relevance and the availability of experimental data for comparison.

Direct solubility calculations offer a range of varied methodologies. While the direct coexistence method remains computationally costly, recent studies have obtained highly accurate results for simple alkali halides.^157^ Further, recent developments to the chemical potentials route have significantly streamlined computational workflows and opened the door for the successful application to organic molecules.^160,177^ Meanwhile, vastly different methods such as the density of states approach have been applied to organic molecules with reasonable success.^146^ Despite these improvements and successes, direct solubility calculations remain computationally intensive and offer limited accuracy and generalizability.

### 3.2 Indirect Calculation of Solubility

#### 3.2.1 Thermodynamic Cycles

Intrinsic solubility can be defined via the Gibbs free energy of solution as

18 $$\Delta G_{sol}^{*}=-RT\ln\left(S_{o}V_{m}\right)$$

where Δ*G*_sol_^*^ is the free energy change of transferring a molecule from the crystalline phase to aqueous solution under standard conditions, R is the molar gas constant, T is the temperature, *S*_*o*_ is the intrinsic solubility in moles per liter and *V*_*m*_ is the molar volume of the substrate crystal. The activity coefficient for the solute in solution is assumed to be unity. The Ben-Naim definition,^183,184^ which describes the transfer of a molecule between two phases at a fixed center of mass in each phase, is used as the standard state, indicated by the superscript *.

The most obvious route with which to calculate intrinsic aqueous solubility is by computing the Gibbs free energy of solution, Δ*G*_*sol*_. The direct prediction of solution free energy is uncommon, however, as an accurate prediction cannot be easily made from a single simulation. As a result, solution free energy is often determined indirectly through several calculations.

Measurement of solution free energy can be achieved through two separate thermodynamic cycles: the fusion and sublimation cycles. The fusion thermodynamic cycle consists of the transfer of a molecule from crystal to solution via a super cooled molten phase, in which a normally solid solute is considered to behave as a liquid. This hypothetical state cannot be reproduced experimentally, and is introduced to allow for the separation of solid phase interactions from solvated interactions. Alternatively, the sublimation cycle follows a molecule through the gas phase to solution, and is accessible through both experiment and computation.

19 $$\Delta G_{sol}^{*}=\Delta G_{fus}^{*}+\Delta G_{mix}^{*}=\Delta G_{sub}^{*}+\Delta G_{solv}^{*}$$

Care must be taken when combining calculations from separate simulations to obtain the free energy of solution or intrinsic solubility. A consistent methodology is required throughout this process to minimize the introduction of error between calculations.

In the following subsections, methods to calculate sublimation, fusion and solvation free energies are discussed separately, before their combination to predict solubility is described.

#### 3.2.2 Sublimation

The indirect modeling of solution free energy through the sublimation thermodynamic cycle sees a given molecule transition from crystal to solution via an isolated state. Of the two steps involved in this process, sublimation and solvation, measuring sublimation and its associated free energy has historically been the more difficult. Experimentally, this difficulty arises due to the fact many approaches, such as calorimetric methods, typically only yield a sublimation enthalpy.^185^ Related thermodynamic properties, such as the sublimation entropy, are often back-calculated from experimentally obtained data. This is not always done, however, leading to an incomplete set of properties for many molecules reported in the literature, which can result in the pairing of data from different experimental procedures and may contribute to any experimental noise observed.^186^

Informatics based approaches, such as QSPR models, can provide relatively accurate predictions of solubility and its related thermodynamic properties when provided with high quality data. However, the benefits of such models are limited by their need for large quantities of reliable experimental data, the predictive capabilities of such models beyond the subset of molecules found within their training data, as well as a lack of an underlying theoretical basis with which to interpret the reasoning behind a given prediction. Beyond the accuracy of experimental data, a lack of relevant descriptors related to solid-state contributions have been found to be another limiting factor in the accurate prediction of solubility for quantitative structure–property relationship models.^187^

Modeling solid state behavior via physics-based approaches generally involves the calculation of enthalpic and entropic contributions from the crystalline and gaseous phases toward the sublimation free energy. The calculation of these properties can take a variety of forms, depending on the level of theory applied and approximations made. These various approaches can be summarized into one of two methodologies: Ψ_*mol*_ and Ψ_*crys*_. The first of these two methods, Ψ_*mol*_, is a model potential based approach in which a combination of force field and quantum mechanical level calculations are made to determine a crystalline lattice energy and thermodynamic contributions from phase specific molecular degrees of freedom. Crystalline lattice energies are typically calculated as the sum of all interactions between a central molecule and those surrounding it. The atom–atom interactions modeled within this system use an intermolecular potential in which the electrostatic term is generated from a Distributed Multipole Analysis (DMA)^188^ of the molecular charge distribution. The lowest energy crystalline structure is then found using this potential. Thermodynamic contributions from phase specific molecular degrees of freedom can be separated into vibrational, rotational and translational molecular motions found in the crystalline and gaseous phases. By making assumptions about the contributions from molecular motion observed in both phases, the equipartition theorem can be applied to calculate each in terms of RT, where R is the molar gas constant and T is the temperature. Intramolecular vibrations are assumed to be equal across gaseous and crystalline structures, which allows for the separation of the crystalline lattice energy into crystalline and gaseous contributions, as well as the calculation of vibrational and entropic contributions through the crystal phonon modes.^189^ From this, sublimation enthalpy can be approximated as Δ*H*_*sub*_ = – *U*_*lattice*_ – 2*RT*, where *U*_*lattice*_ is the crystal lattice energy, and 2RT approximates the contribution of the crystalline and gaseous degrees of freedom to the sublimation enthalpy.^190^ Sublimation entropy can also be determined from this approximation as Δ*S*_*sub*_ = *S*_*crys*_ – (*S*_*gas*_^*trans*^ + *S*_*gas*_^*rot*^), where *S*_*crys*_ is the crystalline phonon entropy, *S*_*gas*_^*trans*^ is the gaseous translational entropy contribution, and *S*_*gas*_^*rot*^ is the gaseous rotational entropy contribution. This 2RT approximation is often applied as the crystal lattice energy is assumed to be the dominant contribution to the sublimation enthalpy. Nonetheless, phonon mode calculations are still needed if one wishes to obtain the entropy and thus the free energy,^191^ or indeed for a more accurate computation of the sublimation enthalpy. Abramov et al. reported intrinsic solubility predictions for benzoylphenylurea and benzodiazepine derivatives using sublimation free energies estimated entirely from crystal lattice energies and the 2RT approximation.^192^ Where more accuracy is required, entropic contributions are included in sublimation free energy calculations, and have been reported for small organic and drug-like molecules in a number of studies.^191,193,194^ There are several inaccuracies associated with the Ψ_*mol*_ approach, namely the 2RT approximation and the use of model potential based calculations. The 2RT approximation applies a universal correction, independent of crystal behavior and structure, to approximate the contribution of molecular degrees of freedom to the sublimation enthalpy. It assumes, among other things, that the molecular vibrations are unaffected by transfer from the crystal to the gas, that they are not coupled to the phonons in the crystal, and that the phonon modes’ contributions follow equipartition. This approximation has been found to be inconsistent between crystals of varying size and rigidity, with particularly significant errors introduced for small molecular crystals.^195–197^

The Ψ_*crys*_ approach by comparison performs rigorous electronic structure calculations on the full crystal structure with periodic boundary conditions. By replacing the model potential and 2RT approximation with quantum mechanical (QM) calculations, including accurately computed phonon mode calculations, one can explicitly represent solid state contributions to the sublimation free energy. This allows more accurate and detailed modeling of the crystal structure to be carried out. The use of quantum mechanics throughout all crystalline calculations enables the consistent use of a single level of theory across all phases, which cannot be done with Ψ_*mol*_. Due to this increased complexity, the Ψ_*crys*_ approach has become increasingly popular and successful recently.^198–200^ Several independent studies have been reported which investigate the accuracy of DFT and post Hartree–Fock based calculations,^201–203^ as well as comparisons of QM based phonon mode calculations to model potential based approaches.^199^ An ab initio fragment-based additive scheme has been proposed for approximating the sublimation enthalpies of small molecular crystals at temperatures nearing 0 K,^204,205^ with a more recent application to sublimation pressures.^206^ Blind studies investigating the development of organic crystal structure prediction and current state-of-the-art methods are periodically released.^11^ Most recently, the seventh blind study ran until September 2022 with a relevant publication expected to be released in due course. Overviews discussing the current state of CSP can also be found in the literature.^207^ However, the Ψ_*crys*_ approaches most often used for CSP are designed to correctly order the energies of different polymorphs, hence identifying the most stable crystal structure, rather than to give accurate absolute sublimation free energies.^208^ Recent studies have also highlighted the need for greater accuracy in polymorph ranking methods, which have been reported to produce problematic conformational energies through DFT based delocalization errors.^209,210^ Fowles et al.^199^ showed that the periodic dispersion-corrected density functional Ψ_*crys*_ PBE-TS method, despite its ability to order alternative structures, could not generate accurate absolute sublimation free energies of the kind required for solubility prediction. Thus, it was necessary to use the Ψ_*mol*_ approach to compute the sublimation leg of the thermodynamic cycle corresponding to solubility. Firaha et al. established an experimental benchmark for crystalline polymorph ranking alongside proposing a single point energy correction for use in lattice energy minimization and phonon mode calculations, with phase transition errors below 2 kJ mol^–1^ reported for their benchmark data set.^211^

The sublimation free energy measures the relative energy of a real or hypothetical crystal structure versus the corresponding ideal gas, and its computation is therefore essential to the field of CSP.^195^ This field has made enormous strides in the last 35 years, from its state being described as scandalous by the then editor of *Nature*,^212^ albeit as a provocative opening line to a broadly constructive article. As evidenced by the periodic “Blind Tests” where the CSP community is challenged with a selection of newly solved and unpublished structures,^11^ the field has progressed to reach an encouraging level of success and accuracy in predicting many organic crystal structures, especially those with less conformational flexibility. Up to now, indirect calculations of solubility using CSP methods to model sublimation have generally been attempted only on compounds with known crystal structures. However, since nearly 90% of polymorph pairs differ in energy by less than 6 kJ mol^–1^ and around 40% to 50% by less than 2 kJ mol^–1^, equivalent respectively to about 1.0 and 0.4 log S units, the question of first-principles solubility prediction using *predicted* crystal structures arises. These estimated energy differences are of similar size to the errors in a high-quality solubility prediction, and suggest that such an approach may lie on the cusp of feasibility.

#### 3.2.3 Fusion

Through the fusion thermodynamic cycle, a given molecule is transferred from crystal to solution via a supercooled liquid state. This state simulates a normally solid solute as a liquid, and allows for the separation of solid-state and solvated interactions. Thermodynamic models which simulate this transition are commonly used for understanding solid–liquid phase equilibria, such as determining the solubility of a given solute over a broad range of conditions. A wide range of thermodynamic models exist, the simplest of which are fully empirical models which make use of interpolation and extrapolation to predict solubility at different conditions.^213,214^ On the other side, there are the semiempirical or fully theoretical excess Gibbs energy (GE) models, such as NRTL,^215^ UNIQUAC,^216^ UNIFAC,^217^ PC-SAFT,^218^ as well as the general solubility equation (GSE).^119^ GE models typically include parameters that are related to properties important to phase equilibria, such as molecular size and polarity, which makes them more reliable for property prediction over a range of conditions. However, this requires the use of experimentally derived high quality data which may not always be available. For example, melting point and the enthalpy of fusion are needed for solubility prediction and are determined experimentally using conventional differential scanning calorimetry (DSC) or adiabatic calorimetry, although for biological systems fast scanning calorimetry (FSC) is often required due to sample decomposition.^219–221^ As such models are often applied as a high throughput screening process of virtual libraries or early stage discovery when sufficient quantities of compound may not be available, their use as predictive models is limited. Unlike GE models, the general solubility equation can be derived from the fusion thermodynamic cycle, and so bears some similarity to physics-based approaches. The GSE predicts solubility from a compound’s melting temperature and octanol–water partition coefficient (logP), so long as some assumptions are applied to the entropy of fusion. Artursson et al. have investigated the quality of solubility predictions made with the GSE, as well as its entropic assumptions, determining the GSE to give reasonable accuracy.^222^ The general solubility equation cannot readily be applied to virtual molecular libraries however as the accurate prediction of melting temperature continues to be a difficult task, with predictive errors above 30 °C still common.^121,122,223^

The application of molecular simulations to the prediction of amorphous solubility for drug-like molecules has been investigated by Luder et al. in a series of studies.^224–227^ With the use of Monte Carlo simulations and the free energy perturbation (FEP) method, free energies associated with the transfer from gas into water (Δ*G*_*hyd*_) and gas into pure amorphous phase (Δ*G*_*ga*_) can be used to estimate the amorphous solubility of drug-like molecules.

20 $$S_{a}=\frac{\exp\left(-\Delta G_{aw}/RT\right)}{V_{m,a}}$$

where *S*_*a*_ is the amorphous solubility, Δ*G*_*aw*_ is the free energy for transferring a molecule from the amorphous phase to aqueous solution (Δ*G*_*aw*_ = – Δ*G*_*ga*_ + Δ*G*_*hyd*_), and *V*_*m*__,*a*_ is the molar volume of the drug-like molecule in the amorphous phase. The authors determined FEP simulations to be too computationally expensive for routine use with drug-like molecules, and so proposed an approximate theory for determining Δ*G*_*ga*_ and Δ*G*_*hyd*_. This new approach, which they refer to as the simplified response (SR) theory, uses linear response theory and mean field theory to approximate for electrostatic and Lennard-Jones interactions, respectively. Due to a lack of experimentally measured amorphous solubilities, the SR method was benchmarked on data derived from experimental intrinsic aqueous solubility (*S*_*a*_), entropy of fusion (Δ*S*_*m*_) and melting point (*T*_*m*_).

21 $$S_{a}\approx S_{0}\exp\left(\frac{\Delta S_{m}}{R}\ln\left(\frac{T_{m}}{T}\right)\right)$$

Vinutha and Frenkel^228^ used a combination of simulation-based methods to investigate the solubility of amorphous materials. They combined direct coexistence simulations with computation of chemical potential via thermodynamic integration in a study relevant to both glasses and supercooled liquid phases. They noted some significant differences between the numerical predictions of the different approaches, though trends were broadly in line with experiment, including a decrease in amorphous solubility with time.

Amorphous solubilities were obtained with an RMSD of roughly 1 log S unit, although model reparameterisation and the introduction of an empirical correction were necessary for this to be achieved. Predicted values were noted to be sensitive to the choice of force field and atomic partial charges used within the model. Further, experimentally derived data is necessary to calculate intrinsic aqueous solubilities.

The determination of amorphous solubility can be useful for molecules that exhibit poor solubility in their thermodynamically stable form. The higher solubility metastable polymorphs can provide greater bioavailability within drug delivery systems, often achieved through the use of amorphous solid dispersion (ASD). With this approach a drug molecule is dispersed within the matrix of an often water-soluble polymer, which in turn provides greater solubility than can be achieved with the crystalline form. Several recent reviews have discussed the use of ASD systems for drug-like molecules,^229–232^ with a range of other studies having investigated the amorphous solubilities of various compounds.^233–235^ Two physics-inspired semiempirical models, the Hildebrand solubility parameter and Flory–Huggins (FH) interaction parameter, are commonly applied for predicting the solubility of solid dispersions. These approaches assume the polymer acts as a solvent and the amorphous polymorph as the solute in a mixed system, with the favorability of the ASD controlled by the Gibbs free energy of mixing: Δ*G*_*mix*_ = Δ*H*_*mix*_ – *T*Δ*S*_*mix*_. The Hildebrand solubility parameter, δ, describes the energy needed to vaporise one mole of molecules from the liquid phase per unit volume, referred to as the cohesive energy density (CED).^236^ The magnitude of δ therefore gives an approximation of the condensed phase intermolecular forces, which can be related to the enthalpy of mixing as

22 $$\Delta H_{mix}=V_{t}{\left(\delta_{1}-\delta_{2}\right)}^{2}\phi_{1}\phi_{2}$$

where *V*_*t*_ is the total volume of the mixture, and δ and ϕ represent the solubility parameters and volume fractions of the individual components, respectively. To better model individual contributions to the total solubility parameter, Hansen developed a three-dimensional form of the Hildebrand parameter: δ^2^, where δ^2^ = δ_*d*_^2^ + δ_*p*_^2^ + δ_*h*_^2^. The Hansen solubility parameter models solubility explicitly in terms of dispersion forces, *δ*_*d*_, polar forces, *δ*_*p*_, and hydrogen-bonding forces, *δ*_*h*_, which gives greater accuracy for solutions containing polar components. The solubility parameter approach has found use as a screening tool for identifying potential carrier polymers, but however is limited by its purely thermodynamic and semiempirical nature. This approach primarily fails to account for entropic contributions to the free energy of mixing or the impact of kinetic terms on the stability of an ASD, and is often dependent on the availability of experimental data.

The FH theory addresses the lack of entropic contributions seen in the solubility parameter approach by specifically considering the entropic effect of the polymer within a system containing compounds with significant size discrepancies.

23 $$\frac{\Delta G_{mix}}{RT}=n_{1}\ln\phi_{1}+n_{2}\ln\phi_{2}+\chi n_{1}\phi_{2}$$

Here, subscript 1 refers to the solvent, subscript 2 is the solute, ϕ is the volume fraction, n is the number of moles, R describes the molar gas constant, T is the temperature, and χ is the Flory-Higgins interaction parameter. As entropic contributions are included the FH interaction parameter a more detailed description of the thermodynamics of ASD systems can be obtained, however kinetic effects are also not considered by FH theory which will hinder its accuracy for more complex systems. Potential shortcomings in the FH interaction parameter have also been investigated in a series of publications by Anderson.^232,237^ A more extensive discussion of the solubility and interaction parameters can be found in reviews focused on ASD modeling.^230,236,238^

#### 3.2.4 Hydration

The modeling of solvated systems and their associated thermodynamic properties has long been an area of active research, resulting in a wide range of possible approaches for the prediction of hydration free energy. These approaches can generally be separated by the method with which solvent structure is represented, and fall into one of two categories: implicit or explicit solvent models. Implicit solvent models do not include any structural characterization of the bulk solvent system, relying only on a continuous dielectric medium to represent solvent; whereas, explicit solvent models include an atomistic representation of both solute and solvent molecules.

##### 3.2.4.1 Implicit Solvation

Implicit solvent models (sometimes also referred to as continuum models) are a popular and widely applied approach for investigating solvent effects in biomolecular and chemical systems due to their relatively low cost and reasonable accuracy against more fundamental molecular simulations. Cramer et al. have developed a series of solvation models, referred to as the “SMx” models.^239–241^ The final model in this series, SM12, was parametrized for several solvation thermodynamic properties, including solvation free energy prediction across neutral and ionised solutes in aqueous or organic solvent systems, achieving average mean unsigned errors (MUE) of 0.5–0.8 kcal mol^–1^ and 2.2–7.7 kcal mol^–1^ for neutral and ionised solutes, respectively. The previous model, SM8, performed comparably when applied to the same data sets, however SM12 was parametrized against a more diverse training set and has been defined for the entire periodic table. A more theoretically rigorous form of the SMx models, SMD, was later released by Marenich et al.,^242^ which treats the bulk electrostatic contribution with the nonhomogeneous Poisson equation (NPE), rather than the generalized Born approximation used by the SMx series. The SMD reportedly performs comparably to the SM8 and SM12 models, with average MUE of 0.6–1.0 kcal mol^–1^ for neutral solutes across aqueous and organic solvents. For ionised solutes, an average MUE of 4 kcal mol^–1^ was achieved, although solvent specific deviations in SFE prediction for anions and cations were reported. The authors claimed that the SMD and SM8 models, at the time of publishing, were more accurate than other available methods. The SMx series of models include solute specific macroscopic parameters, including acidity and basicity parameters, which may not not be readily available for compounds outside those used for model parametrization.^239^ The SMD solvent model has been regularly included in SAMPL blind study submissions,^243,244^ with a range of independent studies focusing on its further development.^245–249^ Alternatively, the polarizable continuum model (PCM) proposed by Tomasi et al.^250^ has led to the development of several different implementations of the PCM framework. The simplest of these implementations, referred to as the conductor-like PCM (CPCM),^251^ includes the conductor-like screening solvation boundary condition, and has been included in a number of studies.^252–254^ A correction of the polarization charge densities is included within CPCM, by introducing a scaling factor *x* for the solvent dielectric constant, ε: *f*(ε) = ε – 1/ε + *x*. An integral equation formalism of the polarizable continuum model (IEF-PCM) was introduced by Cances et al., implementing a more sophisticated treatment of the solvation boundary condition by taking into account anisotropic or isotropic dielectric continuum solvation.^255,256^ IEF-PCM has found success as a relatively accurate model in the prediction of solvation free energy, and has been included in a number of SAMPL submissions.^257–260^

A very similar approach to CPCM, the conductor-like screening model (COSMO), was independently developed by Klamt et al.,^261^ and includes this same polarization charge density correction. The scaling factor used in both methods typically differs, however, with a value of 0.5 used for COSMO.^262^ The primary benefit of using CPCM or COSMO over the original PCM, is due to the introduction of the boundary condition, which eliminated outlying charge errors caused by solute electron density expanding out beyond the solute cavity.^262^ The COSMO approach has since been extended beyond the typical polarizable continuum solvation procedure with the COSMO model for real solvent (COSMO-RS).^263^ This model introduced a statistical mechanics treatment of interacting surfaces, and so is not limited in its description of the solvated environment, as is the case for any model with the polarizable continuum procedure. COSMO-RS has been directly compared to the SM8 solvent model, using the same data set of 2346 solute–solvent pairs across 91 solvents on which the SM8 model was parametrized and tested.^264^ The findings of this study concluded that the COSMO-RS procedure was capable of solvation free energy predictions to within 0.48 kcal mol^–1^ of experimental values, compared to 0.59 kcal mol^–1^ with SM8, without a need for experimental or adjustable parameters. COSMO-RS holds several advantages over more conventional implicit solvent models, enabling its application in mixed solvent and variable temperature systems. Solvent specific parametrization is not needed in the COSMO workflow, with solute and solvent molecules treated equally. Second, QM calculations are performed on a molecule-by-molecule basis, and are not necessary across different solvents. More detailed descriptions of the COSMO and COSMO-RS concepts are available in the literature.^265^ A particular drawback of COSMO-RS, however, is its inability to model the solvent specific response to solute behavior, and so prevents the measurement of properties that can be readily investigated by other continuum models.^262^ This drawback was corrected for with the introduction of the direct COSMO-RS (DCOSMO-RS) method, which replaces the solvent dielectric response with the calculated solute surface polarization charge density.^266^ The COSMO series of models have become a commonly applied tool for solvated phase property investigation beyond solvation free energy,^267,268^ such as phase equilibria,^269–271^ chemical process optimization,^272^ solvent screening^273^ and within drug discovery screening processes.^274,275^ There are drawbacks to the application of implicit solvent models, however, despite the clear advantage of low computational cost. An implicit description of bulk solvent, including important solvent–solute interactions and solvent reorientation, are questionable approximations.

##### 3.2.4.2 Explicit Solvation

Explicit solvent models offer a more rigorous approach to modeling solvated systems than implicit methods, as they more closely represent the underlying physical phenomena. A fully atomistic representation of both solute and solvent enables the consideration of specific solute–solvent interactions and solvent configuration that is not possible with a purely continuum based approach. Explicit solvent models are commonly applied to the prediction of solvation free energy through alchemical free energy methods, such as thermodynamic integration (TI) or free energy perturbation (FEP), applied within atomistic molecular dynamics (MD) or Monte Carlo (MC) simulations. Due to their rigorous theoretical basis, molecular dynamics and Monte Carlo simulations have become a popular molecular modeling approach for investigating biological and chemical systems of interest. Alchemical free energy methods compute solvation free energy through a series of nonphysical intermediary states, which simulate the movement of a solute from vacuum to solution, or vice versa. Further details on their use can be found in the literature.^276,277^ Free energy calculations are routinely performed on a variety of molecular systems and processes, such as, protein folding, protein–ligand binding, and small molecule movement across membranes, among other biologically and chemically relevant systems. Sherman et al. reported MD/FEP calculated solvation free energies with an average unsigned error of 1.10 kcal mol^–1^ for a data set of 239 drug-like molecules.^278^ The authors tested several different force fields and water models, concluding that OPLS_2005 and the SPC water model provided the most accurate results, although semiempirical charge assignment was needed for solute molecules containing particularly polar groups. Sadowsky and Arey proposed a combined QM/MM method, involving additive contributions from both levels of theory.^279^ Ab initio molecular dynamics (AIMD) calculations of ion hydration free energies have been reported in several studies. Leung et al. reported hydration free energies for four monovalent ions to within 4% of experimental measurements through AIMD/TI based calculations.^280^ With this approach, individual thermodynamic integration procedures were necessary for each ion investigated. A similar investigation was carried out by Chaudhari et al. for divalent metal ions, with a comparison of quasi-chemical theory and force field based free energy calculations.^281^ Li and Wang developed ionic pairwise potentials with the adaptive force matching (AFM) method and combined QM/MM calculations, without the need for empirical parametrization.^282^ These AFM models could predict hydration free energies for salts to within 6% of experimental references. Mobley et al. have discussed the use of polarizable iterative atomic charges for biomolecular systems in which the molecular environment undergoes a change in polarization.^283^ With this approach, atomic charges were derived from electronic structure calculations and combined with GAFF bonded parameters. Hydration free energies were predicted to within 2 kcal mol^–1^ of experiment for a data set of 613 organic molecules through free energy perturbation calculations. Fully polarizable force fields, such as AMOEBA,^284^ have received a lot of interest for their inherent ability to respond to dynamic environmental changes during simulations which is not possible with traditional pairwise models.^285–287^ Fully atomistic molecular simulations offer a viable alternative to implicit continuum based approaches, however, the explicit representation of complex systems comes at a far greater cost, often requiring significant time investment to model even a modest number of systems.

##### 3.2.4.3 Reference Interaction Site Model

A range of solvation models have been developed from the integral equation theory (IET) of molecular liquids, offering a viable alternative to expensive fully atomistic simulations and approximate continuum models. IET based models include an explicit representation of solute–solvent interactions via an atomistic force field. Unlike molecular simulation methods, however, the solvated system is described through a set of correlation functions, enabling the efficient computation of solvation structure and thermodynamics from statistical mechanics. A detailed review of the molecular integral equation theory and its applications is available in the literature.^9^ Some of the most recent developments have involved the reference interaction site model (RISM) and the closely related molecular DFT approach. The latter is based on classical DFT and a six-dimensional representation of positional and orientational solvent density. Recent studies on the MDFT theory have shown the method to accurately predict hydration free energies to within 1 kcal mol^–1^ of experiment with an average computation time of 2 CPU minutes per molecule.^288–291^ Although several IET based methods have been developed, only the reference interaction site model (RISM) will be discussed in detail here as it is the most widely implemented approach and the only one that has been used to predict solubility.

The RISM theory uses a simplified form of the high-dimensional molecular Ornstein–Zernike (MOZ) equations to model solvent density distribution around a solute molecule through a set of correlation functions. Solvation free energy predictions are made analytically using one of several available free energy functionals. From the RISM framework, two distinct methods have emerged. The simplest of these is 1D-RISM,^292^ in which the MOZ equations are approximated as a set of one-dimensional integral equations. 1D-RISM is rarely used in its common form for quantitative calculations of solvation thermodynamics as many of its free energy functionals fail to accurately predict the energetic parameters of chemical systems.

These functionals, such as the Hyper-Netted-Chain (1D-RISM/HNC)^293^ and Kovalenko-Hirata (1D-RISM/KH)^294^ models, are too inaccurate for routine use and typically achieve absolute prediction errors above an order of magnitude from experimental values.^295^ More accurate models have since been developed, including the Gaussian Fluctuations (1D-RISM/GF)^296^ and partial wave (1D-RISM/PW)^297^ free energy expressions. However, although several studies have since reported reasonable qualitative agreement with experimental data, large predictive errors are still commonly observed for many chemical systems.^298–301^ There have been various efforts to increase the accuracy of 1D-RISM beyond improvements to the RISM theory, such as through the introduction of empirical corrections, or by replacing the free energy functional entirely with a machine learning model. Ratkova et al. introduced a hybrid RISM and cheminformatics model by including empirical corrections to 1D-RISM calculated SFE.^302^ By including correction parameters determined from chemical descriptors, this structural descriptors correction (SDC) model was able to lower the prediction error of small organic molecules in aqueous solution at 298 K to 1.2 kcal mol^–1^. However, the inclusion of data set specific descriptors limits the wider applicability of this approach, with the potential need for reparameterisation when new molecules are introduced. Machine learning based free energy functionals trained on 1D-RISM correlation functions were introduced by Palmer et al.^303^ This method, RISM-MOL-INF, could make accurate predictions of hydration free energy with partial least-squares (PLS) models on a limited data set of small organic molecules. The RISM-MOL-INF process has recently been overhauled, with a more robust model proposed by Fowles et al. This new method replaced the existing machine learning approach with a deep learning convolutional neural network (CNN), and enabled the accurate prediction of solvation free energy across aqueous and organic solvents beyond 298 K.^304,305^

The more commonly used form of the RISM theory is 3D-RISM, which approximates the MOZ equations with a set of three-dimensional integral equations. The 3D-RISM equations relate 3D intermolecular solvent–solute total correlation functions, *h*_*a*_(*r*), and direct correlation functions, *c*_*α*_(*r*),

24 $$h_{a}\left(r\right)=\overset{n_{solvent}}{\underset{\xi =1}{\sum }}\int_{R^{3}}c_{\xi }\left(r-r^{\prime }\right)\chi_{\xi \alpha }\left(|r^{\prime }|\right)dr^{\prime }$$

where ξ and α denote the indexes of sites in a solvent molecule and *n*_*solvent*_ is the number of sites in a solvent molecule. The bulk solvent susceptibility function, *χ*_*ξα*_, describes the mutual correlations of sites ξ and α in solvent molecules in the bulk solvent. This solvent susceptibility function can be obtained from a preparatory 1D-RISM calculation: *χ*_*ξα*_(*r*) = ω_*ξα*_^*solv*^(*r*) + *ρh*_*ξα*_^*solv*^(*r*), where *h*_*ξα*_^*solv*^(*r*) are intramolecular correlation functions, ω_*ξα*_^*solv*^(*r*) are solvent–solvent site total correlation functions and ρ describes the solvent bulk number density.

To solve for *h*_*a*_(*r*) and *c*_*α*_(*r*), *n*_*solvent*_ closure relations are introduced.

25 $$h_{\alpha }\left(r\right)=\exp\left(-\beta u_{\alpha }\left(r\right)+h_{\alpha }\left(r\right)-c_{\alpha }\left(r\right)+B_{\alpha }\left(r\right)\right)-1$$

Here, *u*_*α*_(*r*) is the interaction potential between solute molecule and α solvent site, *B*_*sα*_(*r*) are bridge functionals, β = 1/*k*_*B*_ *T*, *k*_*B*_ is the Boltzmann constant, and T is temperature.

Generally, exact solutions cannot be computed for the bridge functions, and so must be approximated for. The most commonly used closure relationship is the KH closure developed by Kovalenko and Hirata,^306^ which improves upon convergence rates obtained with the Hyper-Netted-Chain closure and prevents a possible divergence of the numerical solution of the RISM equations.

26 $$h_{\alpha }\left(r\right)=\{\begin{array}{ll} \exp\left(\Xi_{\alpha }\left(r\right)\right)-1 & \Xi_{\alpha }\left(r\right)\leq 0\\ \Xi_{\alpha }\left(r\right) & \Xi_{\alpha }\left(r\right)>0 \end{array}$$

where Ξ_α_(*r*) = – *βu*_*α*_(*r*) + *h*_*sα*_(*r*) – *c*_*sα*_(*r*).

There are several approximate functionals available within RISM for the calculation of solvation free energy. These free energy functionals obtain an analytical solution from the total and direct correlation functions. Many of these functionals have been used extensively,^307–309^ but are generally too inaccurate for routine use and provide hydration free energies with a large error from experiment.^310–312^ The KH and GF free energy functionals are provided below.^294^

27 $$\begin{array}{l} \Delta G_{solv}^{KH}=\rho_{\alpha }k_{\beta }T\overset{n_{solvent}}{\underset{\alpha =1}{\sum }}\int \left[\frac{1}{2}{h_{\alpha }}^{2}\left(r\right)\theta \left(-h_{\alpha }\left(r\right)\right)-c_{\alpha }\left(r\right)-\frac{1}{2}c_{\alpha }\left(r\right)h_{\alpha }\left(r\right)\right]dr \end{array}$$

28 $$\begin{array}{l} \Delta G_{solv}^{GF}=\rho_{\alpha }k_{\beta }T\overset{n_{solvent}}{\underset{\alpha =1}{\sum }}\int \left[-c_{\alpha }\left(r\right)-\frac{1}{2}c_{\alpha }\left(r\right)h_{\alpha }\left(r\right)\right]dr \end{array}$$

where *ρ*_*α*_ is the number density of solvent sites α and θ is the Heaviside step function. A strong linear correlation was observed between the error in hydration free energies calculated using the 3D-RISM/GF model and the 3D-RISM calculated partial molar volume. From this observation, Palmer et al. proposed the universal correction (3D-RISM/UC) free energy functional.^313^

29 $$\begin{array}{l} \Delta G_{solv}^{UC}=\Delta G_{solv}^{GF}+a\left(\rho V\right)+b \end{array}$$

Here, *ρV* is the dimensionless partial molar contribution, a is the scaling coefficient and b is the intercept. The values of the scaling coefficient and intercept are obtained by linear regression against experimental data for simple organic molecules. The 3D-RISM/UC model has been shown to give accurate predictions of HFE to within 1 kcal mol^–1^ of experiment, with further applications to the calculation of hydration thermodynamics.^313–317^ Other semiempirical free energy functionals have been proposed, such as the partial wave correction functional for 1D-RISM (1D-RISM/PWC),^300^ or the cavity corrected functional (3D-RISM/CC).^318^ However, neither model has undergone significant validation beyond small organic molecules.

A theoretically rigorous free energy functional was developed by Sergiievskyi et al., initially referred to as the initial state correction, but later changed to the pressure correction (3D-RISM/PC).^319,320^ This functional was originally developed for molecular density functional theory (MDFT), but can also improve calculated solvation free energies with 3D-RISM by correcting for an overestimation in the solvent pressure. A second functional, the advanced pressure correction (3D-RISM/PC+), was also developed by Sergiievskyi et al., and later shown by Misin et al. to be more accurate than the original PC functional for SFE prediction in 3D-RISM.^321^ More generally, advances in the 3D-RISM theory have enabled the investigation of a wide range of systems, such as the influence of molecular orientation,^322^ or solute parameters in the RISM theory,^323^ calculation of HFE for molecular ions,^324^ salting out effects,^325^ protein–water interactions,^326^ as well as the introduction of coarse-grained solvent models.^327^ Hayashi et al. have recently proposed a hybrid approach involving the angle-dependent IET and 3DRISM in which hydration free energies have been reported for large biomolecular systems with accuracies nearing molecular dynamics simulations.^328–330^ 3DRISM derived descriptors have also been used in combination with machine learning to compute solvation free energies in a wide-range of different solvents.^331^

#### 3.2.5 Solubility

This section will include a critical discussion of methods to obtain aqueous solubility, from the sublimation cycle, fusion cycle, closely related methods, and also data-driven approaches. This section does not intend to be an exhaustive list of published methods, but rather a summary of the various approaches. Interested readers are directed toward other reviews covering individual topics,^7,332–334^ including general introductions into the application of machine learning within computational chemistry.^335,336^ Amaro et al. discuss multi scale modeling of biological systems and its potential applications within drug discovery in their thorough review of the topic,^337^ with a recent study investigating its use for gas solubility.^338^ Figure 4 provides an illustration of routes that can be included across a range of physics-based methodologies for solubility prediction.

**Figure 4:**
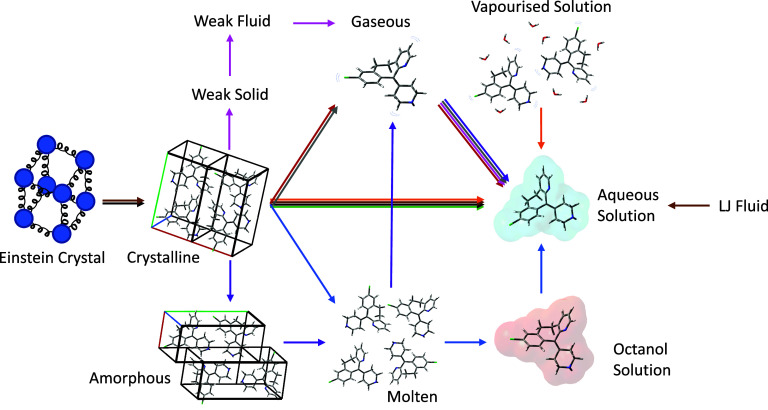
Illustration of routes considered in a sample of physics-based approaches to solubility prediction. Each method links the crystal and aqueous solution through a thermodynamic cycle. Blue = GSE,^119^ Green = Direct Coexistence,^154^ Black = Chemical Potentials,^148,339^ Red = Chemical Potentials,^340^ Pink = Chemical Potentials,^145^ Brown = Chemical Potentials,^341^ Orange = Density of States,^146^ Gray = Simulation Free,^193,199^ Purple = Fusion^224–227^

##### 3.2.5.1 Sublimation Cycle

There have been a wide range of studies investigating aqueous solubility prediction through the sublimation cycle, due in part to the common use of techniques for the calculation of solvation free energy. A molecular simulation approach involving free energy perturbation calculations to estimate the intrinsic solubilities of drug-like small molecules was proposed by Mondal et al.^342^ With this approach, a crystalline lattice is generated through Monte Carlo simulations, and an averaged sublimation free energy determined through a series of five individual FEP+ calculations. Intrinsic solubility is then estimated from the averaged sublimation free energy, and a hydration free energy determined through a single FEP+ calculation. From a data set of 33 small drug-like compounds, a mean unsigned error of 0.6 log units was obtained. No detail was provided for calculated sublimation and hydration free energies, and so it is not known how individual errors contribute to calculated log S values. The authors also reported that this method was successfully integrated as part of a lead optimization study of 103 compounds, identifying a number of analogs with improved aqueous solubility. Schnieders et al. developed a combined MD and stochastic dynamics approach as an alternative to the standard free energy prediction methods used alongside MD simulations.^343^ By combining the AMOEBA polarizable force field with the orthogonal space random walk (OSWR) strategy, Schnieders et al. argued that the crystalline energy landscape could be better replicated than with fixed charge force fields and traditional free energy methods. The OSWR strategy, unlike other free energy methods, is capable of predicting the most favorable crystalline structure, however, a comparison against experimentally observed structures was not included in this study. Similarly to the method proposed by Mondal et al., the AMOEBA/OSWR approach averages calculated sublimation and hydration free energies from five independent simulations. From a data set of seven *n*-alkylamides, hydration, sublimation and solution free energies were reported alongside log S values. However, no experimental hydration or sublimation free energy data was provided. Experimental solution free energies and intrinsic solubilities were available for four of seven *n*-alkylamides, with average errors of 1.1 kcal mol^–1^ and 0.7 log units, respectively. Both of these simulation based approaches are capable of predicting intrinsic solubility to within 1 log unit accuracy, however, a considerable number of simulations are needed for even a single compound, which may limit the applicability of such methods for larger data sets. The AMOEBA/OSWR approach also requires further validation on a larger data set of varied compounds, with a breakdown of component errors, to ensure a cancellation of errors is not favorably leading to accurate log S predictions.

Palmer et al. have reported several methods of predicting intrinsic aqueous solubility for crystalline organic molecules via the sublimation cycle. In an introductory study, a joint direct computation and informatics based approach, using calculated thermodynamic properties combined with molecular descriptors in three-variable linear regression models was tested.^191^ These models were trained on a data set of 34 drug-like molecules, with calculated lattice energies, log P and rotatable bonds per compound included as molecular descriptors. An external test set of 26 molecules was used to validate this linear model, with an RMSE of 0.71 log units achieved. Hydration free energies and sublimation free energies were obtained via QM and CSP calculations, respectively. In this study, a focus was put on using affordable levels of theory with which to calculate thermodynamic parameters. As a result, QM calculations were performed using the B3LYP functional and SM5.4 or SCRF continuum solvent models. Similarly, solid phase calculations included a number of approximations. The 2RT approximation was included in the computation of sublimation enthalpy to avoid costly phonon mode calculations, with the crystal lattice energy determined using a model potential. The entropic contribution to sublimation was assumed to only include gaseous rotational and translational components, and the intramolecular crystalline vibrational contribution. The authors noted that intrinsic solubilities calculated directly from sublimation and hydration free energies did not reach sufficient accuracy, which will be partly a result of the wide ranging approximations used. Palmer et al. later demonstrated that the intrinsic solubility of crystalline drug-like molecules could be estimated from the direct computation of sublimation and hydration free energies.^193^ This approach used the same thermodynamic cycle, but with improved estimates of the sublimation and hydration terms. Model potential based calculations of the crystalline structure were performed with three different functionals (MP2, B3LYP, HF). Hydration free energy calculations were also extended beyond QM/implicit methods with the 3D-RISM/UC model. The sublimation and solvation free energies were calculated in different standard states such that the molar volume of the crystal was not required to compute solubility:

30 $$S=\frac{P_{0}}{RT}\exp\left(\frac{\Delta G_{sub}^{o}+\Delta G_{hyd}^{*}}{-RT}\right)$$

Here, *P*_*o*_ is the standard atmospheric pressure, R is the molar gas constant, T is temperature, superscripts ^*o*^ and * denote the 1 atm and 1 mol/L standard states, respectively. From a data set of 25 compounds, an RMSE of 1.45 log units was obtained through a combination of 3D-RISM/UC hydration and B3LYP sublimation free energy calculations. The model-potentials derived from MP2 and B3LYP calculations provided almost identical sublimation free energy values, with RMSE of 5.63 and 5.66 kJ mol^–1^, respectively, suggesting the source of error for solid phase calculations may derive from the overall modeling strategy and the 2RT approximation rather than the level of theory. In the most recent study, Fowles et al. reported a more rigorous physics-based proof-of-concept that built upon previous work.^199^ Many of the approximations found in the sublimation free energy model were replaced with the explicit calculation of crystal phonon modes through full DFT based PBE-TS calculations. This captured the contributions of the vibrational modes of the crystal and removed the need for the 2RT approximation, allowing for a more accurate estimate of entropic contributions. By combining these improvements with a model-potential based lattice energy, more accurate estimates of sublimation free energy could be obtained than with the more approximate approaches previously reported. This approach was tested on a small data set of three drug-like compounds for which experimental log S were available. The authors also provided experimental hydration free energies and sublimation enthalpies for two compounds, enabling some comparison of methods. From calculated sublimation data, the authors concluded that the use of the 2RT approximation for molecules beyond small organic compounds does not provide reasonable accuracy, with as much as a 17-fold error in solubility when applied to this data set. There was also good agreement between experimental and calculated sublimation enthalpies to within 1.1 kcal mol^–1^. Hydration free energies were evaluated at several levels of theory, with QM calculations involving the PBE, PBE0, and PBE0-DH functionals, as well as with atomistic MD/FEP; from which, the most accurate values were obtained from FEP calculations with errors ranging from 0.38 to 0.86 kcal mol^–1^ of experiment. The authors concluded that combining sublimation and hydration free energies which had been calculated using the highest levels of theory available for either component led to the most accurate log S predictions, as opposed to maintaining a consistent methodology throughout. From this combination of sublimation and hydration free energies, intrinsic solubilities ranging from 0.13 to 1.10 log units were obtained, which the authors noted exceeded the performance of several machine learning models implemented for prediction on this data set. The improvements made to the sublimation free energy routine within this proof-of-concept provide a more rigorous methodology with which to predict intrinsic solubility through physics-based calculations. Figure 5 illustrates the progress that has been made in predicting intrinsic aqueous solubility from a thermodynamic cycle via the vapor using the methods discussed in this section. To further validate this approach, however, it must be tested on significantly more compounds, for which sufficient experimental thermodynamic data is available.

**Figure 5:**
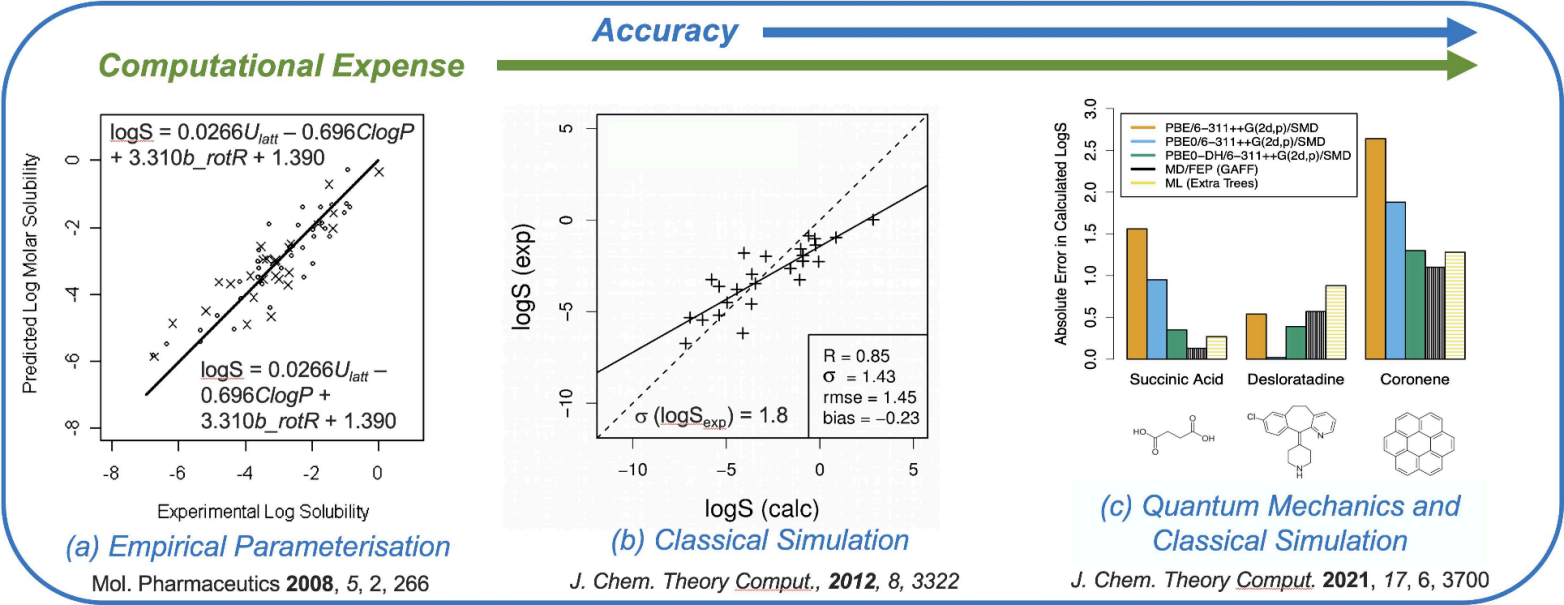
(a) Early methods to predict intrinsic aqueous solubility from a thermodynamic cycle via the vapor required parametrization against experimental solubility data to provide accurate estimates of solubility.^191^ (b, c) Improvements in modeling of sublimation and solvation free energies have enabled physics-based predictions of solubility with improving accuracy at increased computational expense.^193,199^ Reproduced and adapted from references 191, 193, and 199. Copyrights 2008, 2012, and 2021 American Chemical Society.

A straightforward physics-based approach was developed by Abramov et al. for use in guiding the chemical modification of lead compounds with poor solubility.^192^ This approach was intended to replace informatics based models which often rely on training data that does not include a sufficient description of the solid phase contribution.^187^ Here, intrinsic solubility was estimated from sublimation enthalpy and hydration free energy as log *S* ≈ – (Δ*H*_*sub*_ + Δ*G*_*hyd*_), where Δ*H*_*sub*_ = – *U*_*lattice*_ – 2*RT*. The authors noted the decision to approximate sublimation free energy from sublimation enthalpy was driven by the choice to avoid phonon mode calculations, and so is likely to be the main source of error in this approach, alongside errors introduced by the 2RT approximation. This method was tested on two pharmaceutical series with known poor solubility, benzoylphenylurea and benzodiazepine derivatives. As the main goal of this approach was not to explicitly predict intrinsic solubility, no detailed comparison against experimental values has been given, although experimental values have been provided alongside calculated sublimation and hydration free energies. The authors instead conclude that this physics-based approach provides a useful breakdown of sublimation and hydration contributions with which to guide molecular modification for the improvement of poor solubility.

In addition to predicting solubility, physics-based methods such as those discussed in this section provide valuable thermodynamic information about solute transfer from the crystalline phase to vapor and then to aqueous solution. Given that the solubility of a crystalline solute relies on both the properties of the undissolved crystal and the solution itself, the thermodynamic insights afford a deeper understanding not only of which of two molecules is more soluble, but also of the reasons behind the differences in solubility. For example, the Solubility Thermodynamic Profile shown in Figure 6 indicates how changes in solute–solute and solute–solvent interactions, as represented by sublimation and hydration free energies, affect solubility. By contrast, data-driven methods, being statistical rather than rooted in first-principles, supply only a restricted statistical perspective on the core physicochemical mechanisms. Furthermore, as most data-driven models derive solubility predictions based on molecular rather than crystalline structure, they cannot explain or predict varying solubilities across different polymorphs of the same molecule.

**Figure 6:**
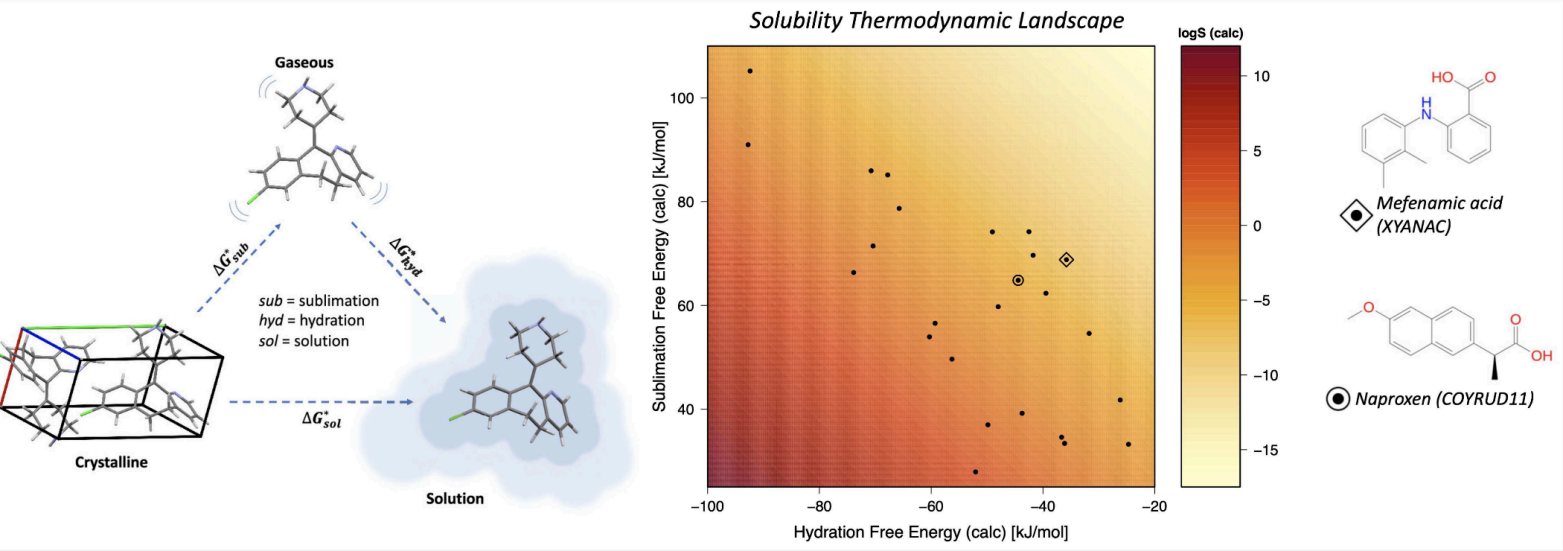
Solubility thermodynamic landscape created from data in Tables 2, 3, and 4 and eq 9 of Reference 193. The solubility of a crystalline organic molecule depends on the chemical potential of the solid and the solution. Solutes with similar solubilities may have very different sublimation and hydration thermodynamics. Reproduced and adapted from ref 199. Copyright 2021 American Chemical Society.

##### 3.2.5.2 Fusion Cycle

A multistage simulation procedure was proposed by Luder et al. for the calculation of amorphous and intrinsic aqueous solubility.^224–227^ With this procedure, solubilities were predicted from the free energy change for transferring a molecule from the pure amorphous phase into aqueous solution. A series of steps are carried out to determine the free energy change associated with the pure amorphous phase, in which the free energy is initially evaluated for a pure melt at 673 K and corrected for the desired free energy change at 298 K with a second set of simulations. The free energy of transfer, amorphous solubility and intrinsic solubility are evaluated with the following equations:

31 $$\Delta G_{aw}=-\Delta G_{va}+\Delta G_{vw}$$

32 $$S_{a}=\exp\left(-\Delta G_{aw}/RT\right)/V_{m}$$

33 $$S_{0}=S_{a}/\exp\left(\Delta S_{m}/R\ln\left(T_{m}/T\right)\right)$$

Here, Δ*G*_*aw*_, Δ*G*_*va*_, Δ*G*_*vw*_ are the free energies of transfer from amorphous to water, solvation in pure melt, and hydration, respectively. Subscript a and 0 refer to amorphous and intrinsic solubility, respectively. Δ*S*_*m*_ is the entropy of melting, *T*_*m*_ is the melting point, and *V*_*m*_ is the molar volume of a given molecule. Two separate methodologies were tested with this procedure: MD/FEP and a semiempirical model involving MC simulations. The semiempirical model included approximations from linear response and mean field approximation theories, with the goal of obtaining accurate solubilites in a shorter time frame than more traditional methods. Across a data set of 46 drug molecules, the authors reported an RMSE of 13 kJ mol^–1^ between amorphous to pure melt free energies calculated with MD/FEP and their semiempirical model. Amorphous and intrinsic solubilities calculated with the semiempirical model were also compared against experimental values for 8 of the compounds in their data set. No statistical analysis was provided, however, reasonable correlation can be noted from plots included in the study. The authors attributed a large source of the error between simulation methods to be a result of uncertainty in the surface tension of the TIP4P water model. Further investigation by the authors concluded that the choice of force field plays a major role in the errors observed, with empirical parameters to correct for systematic errors necessary to achieve reliable predictions.

##### 3.2.5.3 Closely Related Methods

Thompson et al. proposed an alternative approach for predicting intrinsic solubility from hydration free energies and vapor pressures.^344^ Intrinsic aqueous solubilites were compared against calculated values for a data set of 75 liquid solutes and 7 solid solutes through QM calculations involving the SM5.42R continuum solvent model, and the HF, B3LYP or AM1 levels of theory. The relationship between hydration free energy, pure substance vapor pressure, and intrinsic solubility is given as

34 $$S=\left(\frac{P^{{}^{\circ}}}{P}\right)\exp\left(\frac{-\Delta G_{hyd}}{RT}\right)$$

where P is the pressure of an ideal gas at 1 molar concentration and 298 K, and *P*° is the equilibrium pure substance vapor pressure. An additional calculation is performed to obtain the vapor pressure for a given solute from its free energy of self-solvation. From calculations involving the B3LYP level of theory and SM5.42R solvent model, an MUE of 0.36 log units was achieved across the full data set of liquid and solid solutes. Calculated vapor pressures and hydration free energies were also compared against experimental values, with MUE of 0.31 log units and 0.37 kcal mol^–1^, respectively. The authors noted that no additional descriptors for solubility prediction were required, beyond those typically applied for the calculation of solvation free energy. As this method was primarily tested on liquid solutes, there is insufficient data with which to determine how this method would perform on a larger data set of crystalline solutes. As such, additional benchmarking is needed to determine the applicability of this approach.

The COSMO-RS method, originally developed for the prediction of equilibrium thermodynamics, has been expanded to the prediction of solubility for solid compounds. The COSMO-RSol method, proposed by Klamt et al., is a procedure in which fusion free energies are predicted via a multilinear regression model.^345^ This process includes three parameters obtained from a training set of 150 drug-like molecules: chemical potential of the compound in a solvated state, cavity volume and the number of ring atoms per solute. Solubilites are calculated from the fusion thermodynamic cycle, with predicted fusion free energies combined with mixing free energies calculated using COSMO-RS. This method was tested on a data set of 107 pesticide molecules, obtaining an RMSD of 0.61 log units, compared to an RMSD of 0.66 log units for the training set of 150 drug-like compounds. The authors also included a comparison against an HQSAR model, which had been trained on 405 pesticide molecules and achieved a prediction accuracy of 0.72 log units. To the best of our knowledge, COSMO-RSol has not undergone any further testing on additional data sets, however, similar methods have since been reported.^346,347^

Bjelobrk et al. developed an MD approach for modeling the solubility of organic crystals through well-tempered metadynamics simulations.^348^ For a given solute–solvent pair, the free energy difference between the solvated solute and its crystallized state positioned at the edge of the crystal was measured. Simulations were performed at different solution concentrations, and a solubility then determined from the concentration at which the free energy values between states were equal. This method was tested on a single polymorph of urea and naphthalene in a range of organic solvents, with approximately five simulations per solute–solvent pair. The authors reported mole fraction solubilities with experimental errors ranging from 0.0004 to 0.00507, as well as predicted melting points with errors of 14K and 25K for urea and naphthalene, respectively. It was noted that simulation times exceeding 750 ns were necessary to sufficiently sample this dissolution process. Although this method was only tested on organic solvents, it should be trivial to extend toward aqueous solvent. Neha et al.^349^ recently carried out a related molecular dynamics study, using both direct coexistence and chemical potential approaches in their investigations of the solubilities of three urea polymorphs.

##### 3.2.5.4 Data-Driven Approaches

Boobier et al. investigated the performance of various machine learning models for predicting solubility in aqueous and organic solvents.^89^ A range of linear and nonlinear algorithms were each trained on a set of 14 descriptors, of which 11 were derived from quantum mechanical calculations and the remaining 3 were taken from experimental measurements. Five solvent specific data sets were compiled to test this method, covering water, ethanol, benzene and acetone. Two separate data sets were used for water, the first of which included all available data, while the second only included data in the same solubility range as that available for organic solvents. A total of 900 data points were available for the full water data set, 560 for the reduced data set, and 695, 464, and 452 data points for ethanol, benzene, and acetone, respectively. Several results were reported as part of this study. First, nonlinear models all performed comparably, with the extra trees algorithm providing RMSE of 0.54–0.83 log units across all solvent data sets. On average, 70% of solute predictions were reportedly within 0.70 log units of experiment, which is in line with the experimentally measured error. Comparisons were also made against several commonly used prediction tools (AquaSol, EPISuite, and COSMOtherm) using the same evaluation data sets, as well as an external test set obtained from AstraZeneca. The extra trees models outperformed all three benchmarked tools across each data sets. The authors concluded that the availability of high quality experimental data, and the choice of descriptors are more limiting than the chosen model for accurate solubility prediction.

A QSPR based approach was proposed by Abramov, in which solubility is calculated via fusion and mixing free energies predicted from separate models.^187^ Each model used the Cubist algorithm, trained on a large set of Dragon and VolSurf+ descriptors. A set of in-house SMARTS keys were also included as a descriptor. Abramov noted that fusion and mixing free energies were pseudovalues, with fusion values obtained from experimental fusion enthalpies and melting points, and mixing free energies determined using experimental log S and pseudo fusion free energies. For a data set of 62 drug-like molecules, an RMSE of 5.1 kJ mol^–1^ was reported for both predicted fusion and mixing free energies against pseudoexperimental values, while an RMSE of 0.7 log units was obtained for log S. The author determined that no statistical significance was being identified between fusion free energy and descriptors, with an average Pearson correlation coefficient of 0.21, and that a better description of solid state interactions is needed to further improve upon current cheminformatics based approaches.

Vermeire et al. developed a series of deep neural networks capable of predicting a range of thermodynamic properties, including solvation free energy, enthalpy, and solubility of solid solutes at a range of temperatures for organic or aqueous solvent.^350^ Each model was trained on a considerable quantity of experimental data, with upward of 11000 data points used for the solvation free energy and solubility models. Solvation enthalpy and free energy models were initially trained on 1 million and 800000 quantum mechanically calculated values via a transfer learning approach, respectively, before fine-tuning with experimental data. Solubility predictions in a given solvent involved the use of reference values, either obtained from experimental measurements in organic solvent or from predictions of the aqueous solubility.

35 $$\log\left(S_{X,298\;K}\right)=\log\left(S_{ref,298\;K}\right)-\frac{\left(\Delta G_{X,solv,298\;K}-\Delta G_{ref,solv,298\;K}\right)}{R\cdot 298K}$$

Here, subscript ref refers to a reference value, and subscript X is the desired solvent being calculated for. For solubilites predictions beyond 298 K, the dissolution enthalpy at 298 K is introduced as the sum of sublimation and solvation enthalpies.

36 $$\ln\left(\frac{S_{T}}{S_{298\;K}}\right)=\frac{-\left(\Delta H_{sub,298\;K}-\Delta H_{solv,298\;K}\right)}{R}\left(\frac{1}{T}-\frac{1}{298K}\right)$$

Here, Δ*H*_*sub*__,298*K*_ is the sublimation free energy for a given solute–solvent pair, determined from multilinear regression coefficients. Δ*H*_*solv*__,298*K*_ is the predicted solvation free energy. The authors note that the temperature dependence of the dissolution enthalpy can be accounted for through numerical integration for systems nearing the critical temperature of a solvent. For predictions involving aqueous solubility as a reference value, RMSE of 0.89 log units and 1.49 log units were obtained for 1051 data points at 298 K and 4922 data points at a 243–364 K range, respectively. The authors note that when experimental solubilites are available, these errors reduce further, as is the case for experimental data for ethanol solvent, with which errors decrease to 0.29 log units for 785 data points and 0.44 log units for 3071 data points, respectively. This procedure relies on a series of submodels to make accurate predictions of solvation thermodynamic parameters before solubility can be determined. If a single model fails within this series, any subsequent parameters may not be acquirable. More over, although the inclusion of experimental data may improve the quality of some predictions, it will not always be readily available.

With solubility being an important physicochemical property for organic molecules across a variety of fields, many different machine learning approaches have been proposed for its accurate prediction from both physics-based and purely data-driven foundations. As such, new and updated methodologies are regularly released which cannot be covered in detailed. Interested readers are directed toward several more recent articles mentioned here. The relative strengths of graph-based and molecular descriptor featurisation for solubility prediction have been discussed independently by several groups.^98,351^ Similar studies have reported the most effective features from descriptor and fingerprint-based approaches.^352^ Francoeur et al. proposed a SMILES-based molecule attention tranformer (MAT)^353^ model which could obtain accuracies within 1.7 log S units of experiment.^100^ Analyses of the publicly available solubility data commonly used for developing and validating machine learning models has been given by Sorkun et al.^354^ and Llompart et al.^355^ who aim to scrutinize the quality of existing data sets and understand the factors influencing model performance. They conclude that there is not as yet good evidence of advanced neural network models leading to a breakthrough in predictivity for solubility. Ramos et al. developed an ensemble of recurrent neural networks for predicting the solubility of small molecules, which is available for public use via an online application.^356^ Several studies have also proposed generalized approaches for solubility prediction in water and a range of organic solvents.^89,357,358^ Lastly, Llinas et al. begun a series of solubility challenges within the data-driven community to provide insight into the established approaches for solubility prediction. The first challenge^65^ proposed the question of whether a small data set of 100 compounds with high-precision solubility measurements was sufficient to accurately predict the intrinsic solubility of 32 druglike compounds. The findings from this blind challenge^359^ found significant variation in prediction accuracy across the wide range of proposed models, and spurred later discussion on the limitations of existing molecular descriptors used for solubility prediction.^75^ The second challenge provided two test sets of differing quality, one including curated gold standard shake-flask measurements and the other made up of compounds with greater uncertainty, with which contestants were free to apply their own prediction methods to.^5^ The findings from this second challenge showed minimal improvement in prediction accuracy from the use of more complex machine learning algorithms when compared to the first challenge, and emphasized the need for high quality open source data sets with clearer traceability for experimental errors that may be introduced during model development.^6,90^

##### 3.2.5.5 Solubility of Metastable Polymorphs

As discussed in Section 1.3.5, many, perhaps even most, organic compounds are capable of crystallizing into two or more polymorphs with distinct crystal structures and different solubilities. This can have important consequences for industrial applications. A good example is provided by the pharmaceutical sector, where polymorphic form and solubility influence dosage formulation; the problem that Abbott Laboratories had with Ritonavir providing a case in point.^51^

Experimental determination of the solublity of a metastable polymorph is challenging because metastable forms may transform into more stable forms during solubility assays, and because identifying the crystalline form that is present in equilibrium with the saturated solution is difficult. As described in Section 1.5, the cycling between super- and subsaturated solutions in potentiometric solubility assays can sometimes help to identify changes in crystalline polymorphic form,^52^ but these methods are not guaranteed to find any or all of the metastable polymorphs. Recently, there has been growing interest in semiexperimental and computational methods to assess the solubility of metastable polymorphs. The former rely on experimental estimates of the solubility of a stable polymorphic form and the difference in stability of the stable and metastable polymorphic forms, as such they cannot be used for predictions prior to compound synthesis. Semiexperimental methods to assess polymorph solubility are discussed in a recent review.^360^ Conversely, computational methods provide means to predictively assess the solubility thermodynamic landscape. Considering the relationship between sublimation, solvation free energy and solubility that is expressed in a thermodynamic cycle via the vapor (Section 3.2.1), it is evident that the relative solubilities of metastable polymorphs do not depend on the solutes’s solvation free energy. Therefore, calculation of sublimation free energy (or equivalently the free energy or chemical potential of the solid form) is sufficient to estimate the relative solubility of different polymorphs, and when combined with a calculated solubility for a stable form provides the absolute solubility of each polymorph. Despite recent advances in crystal structure prediction (Section 3.2.2), recent studies have highlighted the need for greater accuracy in polymorph ranking methods.^209,210^ Physics-based methods have been used to predict polymorph solubility from both predicted and experimental crystal structures. Neha et al.^349^ used molecular dynamics and both the direct coexistence and chemical potential methods (Section 3.2.5) to compute the solubilities of three urea polymorphs. Mortazavi et al. used crystal structure prediction to assess the relative solubilities of polymorphs of rotigotine leading to the discovery of a new stable form..^361^

## 4 Toward Universal Solubility Prediction Using Physics-Based Methods

Although most physics-based methods have focused on the prediction of intrinsic aqueous solubility under ambient conditions, there is a growing literature on their use for solubility prediction in other conditions, including in nonaqueous solvents or as a function of pH. In this section, recent applications of physics-based methods in these areas are summarized, while areas for future work are highlighted. The current state-of-the-art of data-driven (and semiempirical) approaches will also be described to provide context, but the coverage of those areas is not intended to be exhaustive in that respect.

Since physics-based methods do not need to be (re)­parametrized on solubility data, unlike data-driven methods, their application to nonaqueous solvents, or varying pH should be an opportunity to demonstrate their versatility. In practice though, there has been less work done in these areas than on the prediction of aqueous solubility under ambient conditions. In part this may reflect the importance of water in biological systems, but it also reflects some practical challenges. In moving to nonaqueous solvents, problems may be encountered in accurately modeling the liquid, which requires different intermolecular potentials and, for larger solvent molecules, better configurational sampling. Similarly, the first-principles prediction of pH-dependent solubility requires calculations of acid–base dissociation constants (p*K*_a_), which although well-established in the literature, and implemented in some commercial software, remain computationally expensive and prone to errors for some classes of compounds. The solubility prediction field is expected to benefit from ongoing advances in theory, computation and computing power in related fields in the coming years.

### 4.1 pH-Dependent Solubility

pH can significantly influence the apparent solubility of ionizable compounds, which we define as the total solubility of the neutral and ionised fractions of the molecule. In the literature, a distinction is sometimes made between bulk pH and microenvironmental pH, particularly for systems where localized pH differences exist. Bulk pH refers to the overall pH of the solution, affecting solubility by altering the ionization state of the solute. For instance, weak acids and bases have greater aqueous solubility when ionized, which depends on whether the bulk pH is above or below the p*K*_a_’s of their titratable groups (as discussed in Section 1.3.3). The majority of methods to predict pH-dependent solubility are implicitly targeted at the prediction of bulk pH. Microenvironmental pH represents localized pH variations, often near surfaces or within confined regions, such as near solid particles or in biological environments like cells. These localized pH shifts can cause different ionization states of a compound compared to the bulk solution, leading to enhanced or reduced solubility in these localized regions, particularly if diffusion or mixing of these layers is slow. Such changes in local solubility cause issues in several industries, including pharmaceutical development, where the microenvironmental pH in the solvent close to the surface of a dosage form can influence the local solubility and hence the dissolution rate, absorption and bioavailability. A common strategy to improve dosage forms is to include an additive to modify the pH in the microenvironment to control dissolution..^362,363^ The models used to relate microenvironmental pH, solubility and dissolution rate in the vicinity of a surface (such as a dissolving tablet) fall in three broad categories based on their underlying assumptions. First, the composition of the system at the solid/liquid interace can be assumed to be at thermodynamic equilibrium.^364^ This assumes that saturation takes place and other processes such as dissolution do not provide a rate-determining step. Second, the composition near the surface can be assumed to be diffusion-controlled in which case a diffusional boundary layer adjacent to the dissolving surface exists, and models must account for mass transport through this layer.^365,366^ Third, composition in the vicinity of the surface can be assumed to be controlled by the dissolution rate.^367,368^ In some cases, dissolution rate can be modeled more accurately by modifying the Noyes-Whitney eq (Section 1.4.1) to account for the influence of microenvironmental pH on solubility.^362^ The study of microenvironmental pH is a large and growing field and readers are directed to several excellent review articles for a comprehensive overview of this topic.^362,369^

Although changing the pH can be an effective way to control the total apparent solubility of an ionizable molecule, it does not affect the intrinsic solubility (neutral form only), except indirectly if the choice of buffer has other effects on the solute (i.e., salting in/out, promoting aggregation, etc.). Hence, one strategy to model pH-dependent solubility is to make separate predictions of intrinsic solubility and p*K*_a_ and then to apply the Henderson–Hasselbalch equation or similar relationship. From a physics-based perspective, the acid–base dissociation constant, p*K*_a_, can be estimated from quantum mechanics calculations and a thermodynamic cycle via the gas-phase. Conceptually, this approach can be considered to comprise two steps: (i) a gas-phase calculation of the p*K*_a_; (ii) correction of the gas-phase p*K*_a_ by the SFEs of the neutral and ionised species. The SFE of the proton is normally obtained from standard data tables. Since several different values for it have been published, care must be taken in selecting the appropriate one, considering both the accuracy of the experimental measurements and the manner in which the data is reported, including the standard states and e.g. whether the Galvani potential for bringing the proton from gas-phase into the solvent dielectric has been included.^324^ The other terms are obtained from quantum mechanics calculations, where the solvent environment is commonly incorporated using an implicit continuum model, but can also be included by methods based on integral equation theory or classical density functional theory. Explicit solvent models can also be used, but then molecular dynamics simulations are required to sample the configurational space of the system, thereby dramatically increasing the computational expense of the calculations. Since the added computational expense does not necessarily bring greater accuracy, these approaches are currently less relevant to pH-dependent solubility prediction. A challenge in calculating p*K*_a_ by physics-based methods is obtaining consistency between the SFEs calculated for the neutral and ionised solutes and those obtained from data tables for the proton; since the magnitude of these numbers is typically large, small discrepancies can lead to large errors in p*K*_a_. Nonetheless, reliable predictions with accuracies of ± 0.1 to 0.2 in the value of p*K*_a_ are expected for small organic molecules with single titratable groups.

The same strategy of predicting intrinsic aqueous solubility and p*K*_a_ separately and then using them to estimate pH-dependent solubility can also be used by semiempirical and data-driven approaches. In this case, p*K*_a_ is normally calculated directly from parametrized models rather than indirectly from a thermodynamic cycle via the gas-phase as is done by physics-based approaches. A wide-variety of such parametrized models exist to predict p*K*_a_, including group contribution methods, descriptor-based methods, and graph-based methods. In principle, any of the QSPR approaches that were described in the context of solubility prediction in Section 2 could be retrained to predict p*K*_a_ instead. There is no clear consensus about which set of features or supervised learning algorithm is most accurate, with both traditional statistical models and AI performing well for certain classes of molecules. Several methods are expected to predict p*K*_a_ with an accuracy of ± 0.1 to 0.2 p*K*_a_ units in the value of p*K*_a_. Regardless of whether intrinsic solubility and p*K*_a_ are calculated from physics-based methods, semiempirical approaches or data-driven models, or indeed whether one of the values is obtained from experiment, a combination of the errors from the separate calculations or experiments, and from the assumptions in the Henderson–Hasselbalch equation, can lead to significant errors in solubility pH profiles in unfavorable cases.

The Henderson–Hasselbalch equation can be considered to be an empirical correction to the intrinsic solubility based on the difference between the p*K*_a_ values of the solute and the experimental pH.^370^ For a weak base, it predicts a 10-fold increase in solubility for each unit decrease in pH below the p*K*_a_. In reality, this increase in solubility is limited by the solubility of the salt (which is not infinite) and may be affected by other factors such as ion-pair formation or aggregation of the solute in solution that are not captured by the equation. As a consequence, predictions from the Henderson–Hasselbalch equation do not always agree with experiment, even when intrinsic solubility and p*K*_a_ are known accurately. Bergström et al. demonstrated that pH-dependent solubility profiles deviated from the those predicted by the Henderson–Hasselbalch equation for a data set of 25 amines.^371^ Bonin et al. reported that the Henderson–Hasselbalch equation gave poor predictions of pH-dependent solubility for compounds at Bayer, though data was not provided.^372^ Nonetheless, some successes have been reported. Völgyi et al. showed that the HH equation was valid for six nitrogen containing heterocycles provided that accurate p*K*_a_ and S0 values were employed.^373^ Using p*K*_a_ values derived from the Marvin software, and intrinsic solubility predictions derived from a model trained on 4500 compounds from the PHYSPROP data set, Hansen et al. calculated pH-dependent solubility profiles with a RMSE of 0.79 log units on a set of 22 compounds.^374^ Johnson et al. proposed a hybrid model to predict aqueous solubility that accounted for ionization effects and crystal packing.^375^ By employing a predicted p*K*_a_ and the Henderson–Hasselbalch equation, they were able to calculate the pH-dependent solubility with reasonable accuracy for some example drug-like molecules. The method introduced several interesting ideas, but was limited by the need for an experimental or simulated crystal structure and a reliance on molecular simulation, which increased its computational expense. Several commercial companies provide modules for the prediction of pH-dependent solubility based on some form of the Henderson–Hasselbalch equation, including Percepta from ACD/laboratories^376^ and ADMET predictor software from Simulations Plus,^377^ but the details of these methods are not disclosed.

Due to challenges faced in estimating pH-dependent solubility from calculated intrinsic solubility and p*K*_a_, some data-driven approaches have been developed to predict it directly. These methods can be broadly categorized as those that are trained to predict total solubility at a prespecified pH, and those that are trained to predict total solubility at a user-specified pH. The methods in the first category are often trained on data from a specific experimental assay, using e.g. a given buffer system, which implicitly limits their domain of applicability, but reduces interassay variability. The methods in the latter category accept pH as an input, thereby allowing it to be used to make predictions at different pHs. One of the best examples of both categories is the work by Bonin et al., who collated nearly 300000 data points from 11 solubility assays and showed that a multitask neural network trained to predict solubility at three selected pH values (acidic, neutral basic) gave more accurate predictions than single-task neural networks or Random Forest models based on ECFP-6 fingerprints. The alternative strategy of adding pH as an input variable to the machine learning models led to less reliable models. A multitask neural network is a machine learning algorithm trained to predict multiple properties at the same time (in this instance, solubility at three pHs). During training, the model for each property benefits from information gleaned from the other tasks. Other work on direct prediction of pH-dependent solubility has generally been validated on smaller data sets or homologous compound families. Sun et al. developed a five-variable multilinear regression model to predict pH-dependent solubility, which showed good correlations between calculated and experimental values for 25 molecules.^378^ Galarza and Gomez reported predictions of pH-dependent solubility for 258 compounds using ACD lab software.^379^ Aleksic et al. compared several models developed at Boehringer Ingelheim for the prediction of solubility at pH 2.2, 4.5 and 6.8.^380^ Compared to work on intrinsic solubility prediction, there have been relatively few studies on machine learning approaches to pH-dependent solubility; this is an area where further work is required, which would be supported by the measurement of new accurate pH-dependent solubility data.

### 4.2 Nonaqueous Solvents

Successful physics-based calculation of solubility in organic solvents has already been demonstrated by several groups, but these studies have often focused on small numbers of solutes or limited ranges of conditions, and larger-scale studies comparing different computational methods on polyfunctional solutes (i.e., drugs, pesticides, etc.) are lacking. Nonetheless, the progress made by physics-based methods is encouraging, and several of the existing methods show promising results.

Several of the physics-based approaches that were discussed in Section X have been applied to the prediction of absolute intrinsic solubility in organic solvents. Based on a methodology that was initially applied by Li and co-workers to compute the solubility of naphthalene in water,^148^ Bellucci et al. use the extended Einstein-Crystal methodology to predict solubility of paracetamol in ethanol with good agreement between calculated (0.085 ± 0.014 in mole ratio) and experiment (average 0.0585 ± 0.004).^176^ Although a customized force field was required to model the solution, the parametrization was not carried out against ethanol solubility data, and the associated methodology provides a general approach to force field development for solubility calculations.^381^ Bjelobrk et al. computed solubility of urea in acetonitrile, ethanol, and methanol, and naphthalene in ethanol and toluene.^348^ Since solubility is measured at thermodynamic equilibrium, it is defined as the concentration at which the chemical potentials of the dissolved and undissolved solutes are identical. Here the authors use molecular dynamics simulations of slow-growing crystalline surfaces, rather than the bulk crystalline phase, to probe equilibrium. The energy difference between the solvated solute (A) and its crystallized state at a crystal surface kink site (B), Δ*F* = *F*_*B*_ – *F*_*B*_, was calculated to indicate whether the solution was undersaturated, Δ*F* > 0, at solubility, Δ*F* = 0, or supersaturated, Δ*F* < 0. Equilibrium solubility was identified by extrapolating between the results of simulations carried out at different solute concentrations. The computed solubility values were not in quantitative agreement with experiment, which the authors put down to deficiencies in the GAFF force field, but they were in the right order of magnitude and they did reveal the correct trends between solvents.

Some groups have focused on the calculation of the relative solubility in different solvents rather than absolute solubility. Assuming the precipitate is in the same solid-form in each solvent, the influence of the solid-state can be ignored. The relative solubility of a solute in two solvents can then be assessed from the difference in the solvation free energies of the solute in each solvent. Consequently, calculating relative solubility is a simpler problem than calculating absolute solubility. Liu et al. used alchemical free energy calculations with molecular dynamics simulations to calculate the relative solubilities of 53 solute–solvent pairs representing 8 small organic solutes and 36 solvents. Using alchemical calculations of solvation free energy, the authors report a good correlation between calculated and experimental relative solubility (R^2^=0.85) and a respectable error in ln­(*S*_*A*_/*S*_*B*_) (RMSE = 1.17). Interestingly, poorer predictions of relative solubility were observed when DFT with an implicit continuum model, the SMD method, was used to compute solvation free energy (R^2^=0.1). This observation seems at odds with other studies that have used implicit continuum models successfully for the calculation of solvation free energy in organic solvents,^239^ and solubility.^199^ Notably, Thompson et al. calculated absolute intrinsic aqueous solubilities of liquids and a small number of solids from solvation free energies and vaporisation energies using the SM5.42R model and obtained a mean unsigned error in log_10_ *S* of 0.36.^344^

## 5 Conclusions

Prediction of solubility is essential in fields such as pharmaceutical development, materials science, and environmental chemistry, and ongoing advances in both physics-based and data-driven methods are paving the way for substantial improvements in accuracy and utility. Each approach has its strengths and limitations, and the future of solubility prediction will involve advances in both areas, as well as innovative hybridization of these techniques to overcome their respective challenges.

Physics-based methods, grounded in first-principles, offer a range of potential advantages that make them highly appealing for solubility prediction. These approaches, based on quantum mechanics and/or molecular mechanics calculations, do not rely on parametrization against experimental solubility data, which in theory should allow them to be applied across a broad range of chemical spaces without the constraint of training data availability. Unlike data-driven models, which may struggle to extrapolate beyond the scope of their training sets, physics-based models should theoretically predict solubility in entirely new chemical classes and under different conditions or solvents without requiring extensive reoptimization. Furthermore, these methods yield valuable chemical and thermodynamic insights, such as enthalpic and entropic contributions to solubility, which are useful for optimizing processes like crystallization or solvent selection. Another critical advantage is that these methods can be systematically refined as our understanding of molecular interactions improves or computational power increases, offering a pathway to enhanced accuracy over time.

However, despite their theoretical appeal, physics-based methods are currently less frequently applied in practice compared to data-driven approaches. This disparity stems primarily from several key challenges. First, while physics-based methods provide fundamental insights, they often struggle with accuracy, especially when compared to data-driven methods trained on high-quality solubility data for the given chemical class of interest. When suitable training data is available for the desired chemical domain, machine learning (ML) models can outperform physics-based approaches in terms of accuracy. Additionally, the computational demand of physics-based methods can be prohibitive for some applications, as they often require significant processing power and time to simulate solvation dynamics or crystal structures accurately. Another practical limitation is that physics-based methods frequently depend on the availability of a known or predicted crystal structure, which may not always be accessible, particularly for novel compounds. In this respect, solubility prediction continues to benefit from advances in crystal structure prediction, both in terms of providing more accurate simulated crystal structures, and in terms of improved methods for computing lattice energies and sublimation free energies.

In contrast, data-driven methods, particularly those based on machine learning, have become increasingly popular due to their ability to predict solubility rapidly and with high accuracy, provided that sufficient experimental data is available. These models do best in situations where large, reliable data sets are available for training, allowing them to detect relevant patterns and correlations. However, they can be limited by the scope of the training data, which means their predictions may fail when applied to chemical spaces that deviate from the data they were trained on or to conditions that were not part of the original model development. The key to future success in data-driven methods lies in the continuous accumulation of high-quality solubility data, as well as the development of more sophisticated algorithms that can capture the underlying chemical and physical principles governing solubility.

## 6 Future Perspectives

Looking ahead, the future of solubility prediction will likely involve advances in both physics-based and data-driven approaches, as well as the development of new hybrid methods that combine the strengths of both approaches.

Recent advances in physics-based methods have generally been limited to the prediction of solubility in aqueous media. Further development of these methods to enable solubility prediction in different solvent compositions and under nonstandard conditions would be of great benefit to the field. The curation of a unified set of industrially relevant solutes, each with accurate experimental measurements of solubility, thermodynamic parameters (e.g., Δ*G*, Δ*H* and Δ*S* of solution, solvation and sublimation) and crystal structures for would drive innovation in this area.

One of the key advantages of physics-based methods is that they can be systematically improved based on a clear theoretical framework. As such many of these methods are expected to benefit from ongoing work to improve quantum mechanics and molecular mechanics methods, including but not limited to better density functionals and basis sets and improved force fields or AI potentials. Moreover, as physics-based calculations move from being applied to small rigid organic solutes in water only, and become more routinely used for larger solutes in mixed solvents, the efficiency with which conformational and configurational space can be sampled will become more important. Improvements in this area will be obtained from advances in enhanced sampling methods and computational power.

Since solubility depends on the physical form of the undissolved precipitate, the perfect solubility model would be able to make accurate predictions for all relevant solid forms, including, for example, amorphous and crystalline states, and different polymorphs, salts, and hydrates. Data-driven models are normally trained to predict solubility for one solid form (e.g., the pure crystal) and small changes (e.g., in polymorphic form) are usually ignored. This introduces a limitation to the model and an implicit error in the predictions. Most physics-based methods do take into account the influence of the solid form, but have to rely on a simulation of the solid form when experimental structural data is not available. As such physics-based solubility prediction will benefit from future advances in solid form prediction, especially crystal structure prediction methods.

As physics-based and data-driven approaches continue to develop, an attractive option to reduce their short comings is to combine them. Hybrid models could leverage the accuracy of machine learning where data is available, while using physics-based insights to guide predictions in uncharted chemical spaces or novel solvents. Additionally, machine learning models could be used to improve the efficiency of physics-based simulations, helping to reduce computational costs by identifying the most relevant molecular conformations or improving or replacing force-field parameters. Another promising hybrid approach is to combine data-driven solubility approaches with self-driving laboratories for fully autonomous solubility prediction.^382,383^ Such automated experimentation can accelerate solubility data generation allowing new areas of chemical space to be explored more efficiently.

The ability of physics-based methods to systematically improve as computational power advances offers long-term promise, particularly as high-performance computing (HPC) and quantum computing become more accessible. These developments could help overcome current limitations in computational demand, making physics-based predictions more feasible for larger and more complex systems. Similarly, advances in machine learning techniques, such as transfer learning and active learning, could help expand the applicability of data-driven methods, allowing them to perform better in low-data or out-of-sample scenarios.

Advances in software and numerical computing resources are also required to enable nonspecialists to benefit from these methods. This is especially important for physics-based methods that often require calculations using multiple different techniques and significant domain knowledge. Recent work by companies such as Schrodinger and others show progress in this respect. Applications in pharmaceutical drug discovery and agrochemical industries make this an important area for ongoing development.

Comparing the performance of various solubility methods becomes problematic when each approach is evaluated on a different, and often small, test set. Over the last few decades, two Blind Challenges for the Prediction of Aqueous Solubility that have been organized by the American Chemical Society have helped to benchmark data-driven prediction methods, and drive innovation. The physics-based solubility prediction community would now benefit from its own blind solubility prediction challenge for the validation of physics-based methods from a single, high-quality data set. This experimental benchmark data set would ideally include crystallographic and thermodynamic data in addition to solubility measurements so that all aspects of physics-based predictions could be independently validated. Significant value to current research efforts could be found by combining future blind challenges for physics-based and AI/ML solubility prediction with crystal structure prediction challenges for the same molecules. To reach this goal would require a concerted effort from both computational researchers to bring together related but often distinct communities, and experimental researchers to measure and collate the required physical and thermodynamic data.

In conclusion, while physics-based methods are currently underutilized due to their computational intensity and dependence on structural information, they hold considerable long-term potential for improving solubility prediction, particularly in unexplored chemical spaces or novel environments. Data-driven methods will continue to dominate where sufficient data exists, but their future success will depend on how well they can generalize beyond their training data. By integrating these two approaches, future solubility models will likely achieve greater accuracy, robustness, and versatility, ultimately accelerating progress in drug development, materials design, and other critical applications.

## Abbreviations

ADMET: Absorption, Distribution, Metabolism, Elimination, and Toxicity
AFM: Adaptive Force Matching
AIMD: Ab Initio Molecular Dynamics
B3LYP: Becke, 3-parameter, Lee–Yang–Parr
bRo5: Beyond (Lipinski) Rule of Five
CheqSol: Chasing Equilibrium Solubility
CC: Cavity Correction
CNN: Convolutional Neural Network
COSMO: Conductor-like Screening Model
COSMO-RS: Conductor-like Screening Model for Real Solvents
CPCM: Conductor-like Polarizable Continuum Model
CPR: Chemical Potential Route
CSP: Crystal Structure Prediction
DFT: Density Functional Theory
DL: Deep Learning
DMA: Distributed Multipole Analysis
DOS: Density of States
DSC: Differential Scanning Calorimetry
DTT: Dissolution Template Titration
EECM: Extended Einstein Crystal Method
FEP: Free Energy Perturbation
FSC: Fast Scanning Calorimetry
GC: Group Contribution
GCNN: Graph Convolutional Neural Network
GE: Gibbs Energy
GF: Gaussian Fluctuations
GSE: General Solubility Equation
HF: Hartree–Fock
HSP: Hansen Solubility Parameters
HNC: Hyper-Netted Chain
IEFPCM: Integral Equation Formulism Polarizable Continuum Model
IET: Integral Equation Theory
KH: Kovalenko-Hirata
LLM: Large Language Model
logP: Logarithm of the Octanol–Water Partition Coefficient
MC: Monte Carlo
MD: Molecular Dynamics
MDFT: Molecular Density Functional Theory
ML: Machine Learning
MOZ: Molecular Ornstein–Zernike (equation)
MP: Melting Point
MP2: Møller–Plesset Second Order
MUE: Mean Unsigned Error
NN: Neural Networks
OSRW: Orthogonal Space Random Walk
PC: Pressure Correction
PCM: Polarizable Continuum Model
PC+: Pressure Correction Plus
PLS: Partial Least Squares
PW: Partial Wave
PWC: Partial Wave Correction
QM: Quantum Mechanics
QSPR: Quantitative Structure Property Relationship
RED: Relative Energy Difference
RF: Random Forests
RISM: Reference Interaction Site Model
RMSD: Root Mean Square Deviation
Ro5: (Lipinski) Rule of Five
SDC: Structural Descriptor Corrections
SFI: Solubility Forecast Index
SPC: Simple Point Charge (model)
SPC/E: Simple Point Charge Extended (model)
SR: Simplified Response (theory)
SVM: Support Vector Machines
TI: Thermodynamic Integration
UC: Universal Correction
